# Higher-order assembly of crystalline cylindrical micelles into membrane-extendable colloidosomes

**DOI:** 10.1038/s41467-017-00465-z

**Published:** 2017-09-04

**Authors:** Hongjing Dou, Mei Li, Yan Qiao, Robert Harniman, Xiaoyu Li, Charlotte E. Boott, Stephen Mann, Ian Manners

**Affiliations:** 1 School of Chemistry, University of Bristol, Cantock’s Close, Bristol, BS8 1TS UK; 2 The State Key Laboratory of Metal Matrix Composites, School of Materials Science and Engineering, Shanghai Jiao Tong University, Shanghai, 200240 China

## Abstract

Crystallization-driven self-assembly of diblock copolymers into cylindrical micelles of controlled length has emerged as a promising approach to the fabrication of functional nanoscale objects with high shape anisotropy. Here we show the preparation of a series of crystallizable diblock copolymers with appropriate wettability and chemical reactivity, and demonstrate their self-assembly into size-specific cylindrical micelle building blocks for the hierarchical construction of mechanically robust colloidosomes with a range of membrane textures, surface chemistries and optical properties. The colloidosomes can be structurally elaborated post assembly by in situ epitaxial elongation of the membrane building blocks to produce microcapsules covered in a chemically distinct, dense network of hair-like outgrowths. Our approach provides a route to hierarchically ordered colloidosomes that retain the intrinsic growth activity of their constituent building blocks to permit biofunctionalization, and have potential applications in areas such as biomimetic encapsulation, drug delivery, catalysis and biosensing.

## Abstract (web summary)

Functional nanoscale objects can be prepared via crystallization-driven self-assembly of diblock copolymers. Here the authors show the self-assembly of crystalline block copolymers into size-specific cylindrical micelles for the hierarchical construction of mechanically robust colloidosomes with a range of membrane textures.

## Introduction

Self-assembly and stabilization of colloidal particles at oil/water droplet interfaces is an attractive bottom-up approach to the fabrication of functionally advanced polymer and inorganic microcapsules known as colloidosomes^1–6^. Colloidosomes have diverse applications in areas such as micro-encapsulation and controlled delivery^7–10^ and catalysis^11–14^, and have been exploited as synthetic protocells exhibiting membrane-gated enzyme reactivity^15^, artificial cytoskeletal assembly^16, 17^, growth and division^18^, and chemical signalling^19^. In each case, colloidal particles with appropriate wettability spontaneously assemble at the oil/water interface to produce Pickering emulsions that are subsequently stabilized by gelation or polymerization of the solvent-filled core, or alternatively, by thermal annealing or chemical crosslinking of the closely packed particulate shell, to produce mechanically robust colloidosomes^1, 15–19^.

A wide range of inorganic and polymeric building blocks have been used for colloidosome fabrication. Typical examples include inorganic colloids consisting of hydrophobically modified silicas^20, 21^, titania^22^, a metal–organic coordination compound^23^, clay platelets^19, 24, 25^ and graphene oxide sheets^26^, as well as numerous modified polystyrene latexes^1, 2, 27, 28^, copolymers^29, 30^ and protein nanofibrils^31^. In many cases, the use of spherical particles or platelets leads to close packing in the colloidosome shell to produce a semipermeable membrane. Recently, highly anisotropic particles of cellulose^32, 33^ and epoxy polymers^34^ have been employed as relatively large and rigid micro-rods for the preparation of Pickering emulsions. In contrast, Armes and colleagues have introduced a new strategy for water-in-oil Pickering emulsion stabilization based on the self-assembly of block copolymer (BCP) nanostructures in the form of worm-shaped micelles with appropriate oil/water wettability^35, 36^.

Herein, we develop a complementary approach using chemically transformed poly(ferrocenyldimethylsilane) (PFS)–poly(methylvinylsiloxane) (PMVS) diblock copolymers to prepare size-specific cylindrical micelles comprising a crystalline PFS core and disordered carboxylated PMVS corona by a seeded growth process known as living crystallization-driven self-assembly (CDSA)^37–41^. We exploit these specialized BCP building blocks for the hierarchical construction of mechanically robust polymer colloidosomes with a range of membrane textures, surface chemistries and optical properties. Significantly, regions of the PFS core exposed at the termini of the cylindrical micelles remain active to epitaxial elongation on addition of BCP unimers^37, 38^. As a consequence, the crosslinked colloidosome membrane can be structurally and chemically elaborated post assembly by a highly regulated in situ growth process. We use this strategy to generate colloidosomes with hair-like outgrowths consisting of elongated cylindrical micelles with contiguous fluorescent domains or arrays of biotinylated side chains capable of streptavidin binding. Overall, our results provide a step towards the interfacial assembly of colloidosomes that retain the intrinsic growth activity of their constituent building blocks. This approach could open up new routes to bespoke hierarchical structures for use in biomimetic encapsulation, drug delivery, catalysis and biosensing.

## Results

### Construction of hierarchical crystalline colloidosomes

**Fig. 1:**
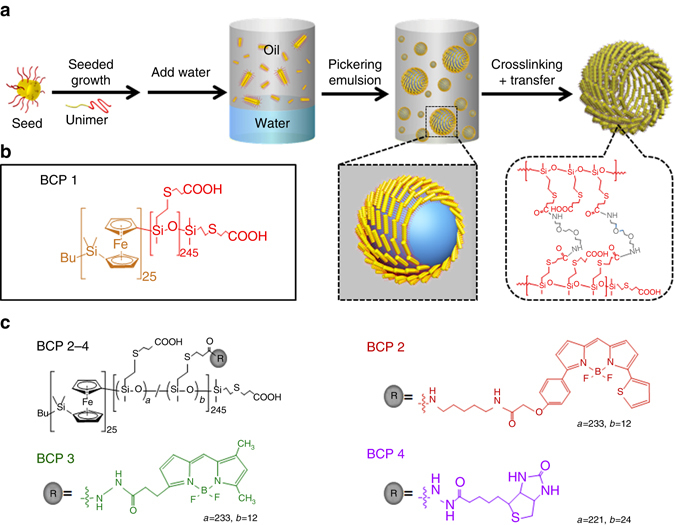
Design and construction of chemically functionalized block copolymer (BCP) colloidosomes. **a** General procedure for formation of colloidosomes. PFS_25_-*b*-PMVSCOOH_245_ (BCP1) unimers undergo self-assembly in 2-ethyl-1-hexanol (EHOH) into core-shell cylindrical micelles of controlled length by a seeded growth process of crystallization-driven self-assembly (CDSA). Mixing the micelle dispersion in EHOH with water or aqueous solutions of molecular cargoes (proteins, dyes and dextran) produces water-in-oil Pickering emulsions, which are stabilized by a membrane of closely packed BCP1 cylindrical micelles (*inset*). Crosslinking the membrane with 2,2ʹ-(ethylenedioxy)bis(ethylamine) (EDEA) followed by removal of the oil layer and subsequent dialysis results in transfer of the colloidosomes into bulk isopropanol or aqueous solution (*inset*). **b** Chemical structure of BCP1. **c** Chemical derivatives of BCP1 containing BODIPY fluorophores (BCP2, *red*; BCP3, *green*) or biotin residues (BCP4) covalently linked to the PMVSCOOH block. Cylindrical micelles of BCP2 and BCP3 are prepared in EHOH by CDSA using BCP1 seeds in the presence of mixtures of BCP1/BCP2 or BCP1/BCP3 unimers

An integrated strategy involving polymer design, interfacial assembly and seeded growth was exploited for the fabrication of colloidosomes comprising a semipermeable membrane of closely packed cylindrical BCP micelles (Fig. 1a). Cylindrical micelles with appropriate levels of wettability were prepared by chemical modification of the diblock copolymer PFS_25_-*b*-PMVS_245_ (*M*_n_ = 27,250 g mol^−1^, polydispersity index (PDI) = 1.18; Supplementary Fig. 1)^38, 39^. Thiol-ene photoirradiation of PFS_25_-*b*-PMVS_245_ in the presence of 3-mercaptopropionic acid and 2,2-dimethoxy-2-phenylacetophenone (DMPA, photoinitiator) was used to produce PFS_25_-*b*-PMVSCOOH_245_ (BCP1; Fig. 1b, Supplementary Fig. 2 and Methods), which consisted of a hydrophobic and crystallizable short PFS core-forming block along with a longer PMVS corona-forming domain comprising hydrophilic carboxylic acid groups. In some experiments, around 5% of the carboxylic acid groups were functionalized with different 4,4-difluoro-4-bora-3a,4a-diaza-s-indacene (BODIPY) fluorophores (BCP2, *red*; BCP3, *green*; Fig. 1c) or 10% with biotin side groups (BCP4; Fig. 1c, Supplementary Fig. 2 and Methods). Dissolution of BCP1 in 2-ethyl-1-hexanol (EHOH) followed by heating to 70 °C for 1 h and subsequent cooling to room temperature produced cylindrical micelles of BCP1 with polydisperse lengths (*L*_n_ = 3870 nm, *L*_w_/*L*_n_ = 1.23, where *L*_n_ and *L*_w_ were the number- and weight-averaged contour lengths, respectively, see equations 1 and 2 in Supplementary Methods). The dispersions were then cooled to 0 °C and sonicated for 1 h to afford a solution of short BCP1 micelles of narrow size distribution (*L*_n_ = 43 nm, *L*_w_/*L*_n_ = 1.05, Supplementary Fig. 3). The short micelles were subsequently used as monodisperse particles for colloidosome assembly, or as seeds for the epitaxial growth of BCP1 cylindrical micelles with narrow length distributions (*L*_n_ = 320 and 1129 nm, and *L*_w_/*L*_n_ = 1.06 and 1.02, respectively; Supplementary Fig. 3), which were then employed as colloidosome-building blocks. Seeded growth was controlled by varying the concentrations of the BCP1 seeds and BCP1 unimers in an EHOH or isopropanol dispersion^37^. This process was also adapted for the epitaxial growth of size-specific fluorescent BCP1/BCP2 and BCP1/BCP3 cylindrical micelles (*L*_n_ and *L*_w_/*L*_n_ = 756 nm and 1.02, and 789 nm and 1.02, respectively) by addition of a mixture of unimers to a dispersion of the BCP1 seed micelles (Supplementary Methods).

### Interfacial self-assembly of size-specific cylindrical micelles

Water-in-oil Pickering emulsions were prepared by vortexing at room temperature small amounts of Milli-Q water or aqueous solution with an EHOH dispersion of size-specific BCP1 cylindrical micelles. Optical microscopy (OM) images indicated that stable emulsions were produced using BCP1 building blocks of variable controlled length (*L*_n_ = 43, 320 and 1129 nm) provided that the water/oil volume fraction (*ϕ*_w_) was between 0.03 and 0.20 (typically 0.05), and BCP1 concentrations were between 0.25 and 4.0 mg mL^−1^. The average diameter of the water droplets increased as *ϕ*_w_ was increased at a constant BCP1 concentration of 0.5 mg mL^−1^ (Supplementary Figs 4–6). This was consistent with the stabilization of a decreased oil/water interfacial area, and in agreement with previous studies on Pickering emulsions^1, 15, 27^. Interestingly, increasing the length of the BCP1 cylindrical micelles also stabilized larger emulsion droplets at constant values of *ϕ*_w_ = 0.20 and BCP1 concentration of 0.5 mg mL^−1^(Supplementary Figs 5 and 6). For example, the droplet diameter increased from 3.8 to 10.8 μm when the length of the cylindrical micelle building blocks was increased from 43 to 1129 nm. Overall, by combining changes in micelle length, water/oil volume fraction and BCP1 concentration we were able to prepare Pickering emulsion droplets ranging from 1.8 to 10.8 μm in average diameter.

**Fig. 2:**
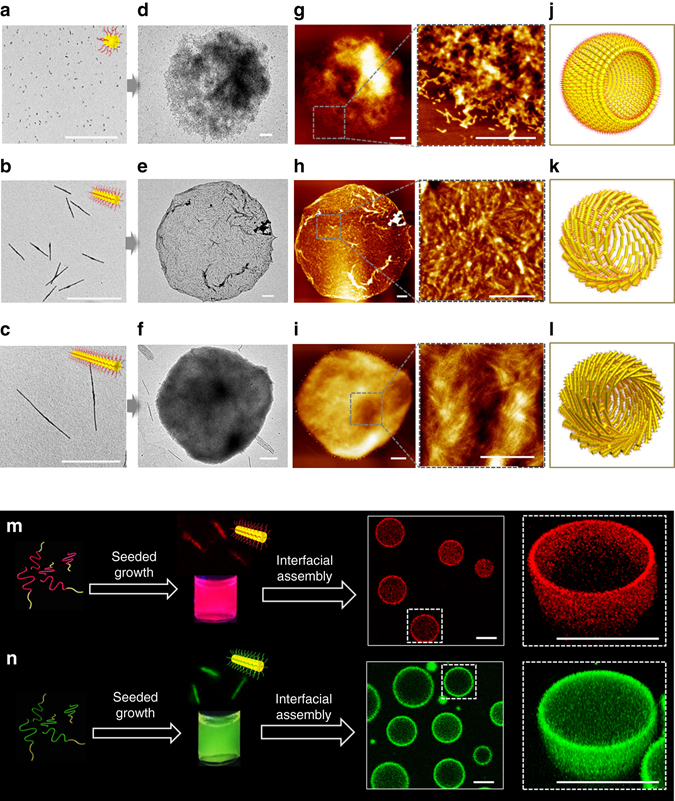
Interfacial self-assembly of size-specific supramolecular building blocks into Pickering emulsions. **a**–**c** TEM images showing building blocks based on 43 nm-sized micellar seeds (*L*_n_ = 43 nm, *L*_w_/*L*_n_ = 1.05) (**a**), and 320 (*L*_n_ = 320 nm, *L*_w_/*L*_n_ = 1.06) (**b**) or 1129 nm-long (*L*_n_ = 1129 nm, *L*_w_/*L*_n_ = 1.02) (**c**) cylindrical micelles. *Insets* depict the crystalline PFS core (*yellow*) and disordered PMVSCOOH corona (*red*). **d**–**f** Corresponding TEM images of air-dried water-in-EHOH Pickering emulsion droplets prepared from the building blocks shown in **a**–**c**; in each case an intact non-crosslinked membrane of closely packed BCP1 micelles is assembled at the oil/water droplet interface. Note the different membrane textures and alignment of the 320 and 1129 nm-long micelles parallel to the membrane surface. **g**–**i** Pairs of large area and corresponding high-resolution AFM images showing membrane textures associated with the microstructures presented in **d**–**f**. Dashed lines and boxes indicate areas selected for high-magnification imaging. **j**–**l** Corresponding graphical representations. **m**, **n** Preparation of fluorescent Pickering emulsions based on BCP1 seed-mediated CDSA of mixtures of BCP1 and fluorescently labelled BCP2 (**m**) or BCP3 (**n**) unimers. Interfacial assembly of the doped cylindrical building blocks (*L*_n_ = 756 nm and *L*_w_/*L*_n_ = 1.02 for *red* cylinders, *L*_n_ = 789 nm and *L*_w_/*L*_n_ = 1.02 for *green* cylinders) in EHOH/water mixtures gives rise to intact hollow spheroids with *red* or *green* fluorescent membranes as shown by the confocal fluorescence microscopy images recorded under UV irradiation at 365 nm. Corresponding *Z*-stack images of the microcapsules identified by the *dashed boxes* are shown on the far *right*. The fluorescent cylinders were taken from separate regions of the CLSM images. *Scale bars* in **a**–**l**, 1 µm. *Scale bars* in **m** and **n**, 5 µm

The above observations indicated that the wettability of the PFS_25_-*b*-PMVSCOOH_245_ diblock copolymer nanostructure was sufficient to drive preferential adsorption of the BCP1 cylindrical micelles at the water/EHOH droplet interface. Transmission electron microscopy (TEM) images of air-dried samples showed intact electron-dense hollow spheroids corresponding to partially collapsed emulsion droplets comprising a continuous shell of BCP1 cylindrical micelles aligned parallel to the membrane surface (Fig. 2a–f). Increasing the length of the cylindrical building blocks increased the structural integrity of the membrane with respect to collapse of the microstructures in the TEM. Individual particles of the seed micelles could be discerned in the collapsed membrane (Fig. 2d), whereas the cylindrical micelles remained closely packed into a densely interwoven thin continuous shell (Fig. 2e, f). We attribute aggregation of the cylindrical micelles to drying artefacts incurred during sample preparation, or hydrogen bonding between pendent –COOH groups. Individual micelles were apparent in high-magnification TEM and atomic force microscopy (AFM) images and their contour lengths were compatible with the lengths of the size-specific building blocks used to assemble the colloidosomes. AFM images were consistent with the membrane textures displayed by TEM (Fig. 2g–i), and line analyses indicated that the heights of the collapsed and often crumpled microstructures were between 20 and 70 nm independent of which building block was used (Supplementary Fig. 7). Given that the width of a single BCP1 cylinder was ca. 8 nm (Supplementary Fig. 8), the similarity between the measured minimum height of the collapsed colloidosomes (ca. 20 nm) and a superimposed double layer of cylindrical micelles oriented parallel to the oil/water interface suggested that the colloidosome membrane consisted of a robust monolayer or few multilayers of closely packed BCP1 nanostructures (Fig. 2j–l). In this regard, AFM elastic modulus mapping gave an average value of 1.67 GPa for the Young’s modulus of the BCP1 membrane (Supplementary Fig. 9), which was comparable to the modulus of bulk PFS homopolymer^42^ (ca. 0.78 GPa), and exceeded the values reported for conventional polymersomes by ~100-fold^43^.

We developed the above procedures to prepare fluorescent semipermeable microcapsules of PFS_25_-*b*-PMVSCOOH_245_ (Fig. 2m, n). For this, we used BCP1-mediated seeded growth in isopropanol to prepare size-specific BCP1 cylindrical micelles doped with ~12.5 wt% of red or green fluorescent BODIPY-functionalized BCP2 or BCP3 unimers, respectively (Supplementary Fig. 10). The fluorescent micelles were ~750 nm in length and uniform (*L*_w_/*L*_n_ = 1.02 for both red and green fluorescent micelles; Supplementary Fig. 11), and showed absorption/emission bands at 601/620 and 509/514 nm for the BCP1/BCP2 and BCP1/BCP3 nanostructures, respectively (Supplementary Fig. 12). Confocal laser scanning microscopy (CLSM) images showed well-defined Pickering emulsion droplets with red or green fluorescence associated with the interfacial assembly of a shell of BCP2- or BCP3-doped cylindrical micelles at the oil/water interface (Fig. 2m, n), together with a small amount of free cylindrical micelles in the water core. Introduction of the fluorescently tagged cylindrical micelles not only allowed direct observation of the interfacial assembly of the micelles but also provided a probe for studying post-assembly modifications to the membrane structure and composition of PFS_25_-*b*-PMVSCOOH_245_-based colloidosomes (see below).

Cross-linking of the size-specific BCP1 cylindrical micelles after interfacial assembly was achieved by amidation of the coronal carboxyl groups using 2,2ʹ-(ethylenedioxy)bis(ethylamine)^44^ (Methods). The resulting colloidosomes could be transferred into aqueous media by dialysis against a series of water/isopropanol mixtures with increasing water volume fractions to produce semipermeable intact microcapsules (Supplementary Fig. 13). CLSM images of fluorescein isothiocyanate (FITC)–dextran-encapsulated colloidosomes recorded after transfer into a water/isopropanol mixture (volume ratio = 4:1) indicated that independent of the length of the cylindrical micelle building block entrapped polysaccharide macromolecules with average molecular weights (*M*_w_) of 4.4 or 10 kDa were able to freely diffuse through the BCP1 membrane, whilst those with a *M*_w_ value 150 kDa were retained within the aqueous interior (Supplementary Fig. 14). Interestingly, passive membrane diffusion of a 70 kDa dextran was observed for colloidosomes prepared from shorter cylindrical micelles (*L*_n_ = 43 or 320 nm) but not for microcapsules comprising building blocks that were 882 nm in length (Supplementary Fig. 14). In the latter case, changes in osmotic pressure did not alter the size or shape of the crosslinked colloidosomes, which remained structurally robust under these conditions. A control experiment involving a mixed solution of crosslinked BCP1 colloidosomes and FITC–dextran indicated that there is no specific interaction between the cylindrical micelles and FITC–dextran molecules (Supplementary Fig. 15).

### Membrane engineering of crystalline colloidosomes

**Fig. 3:**
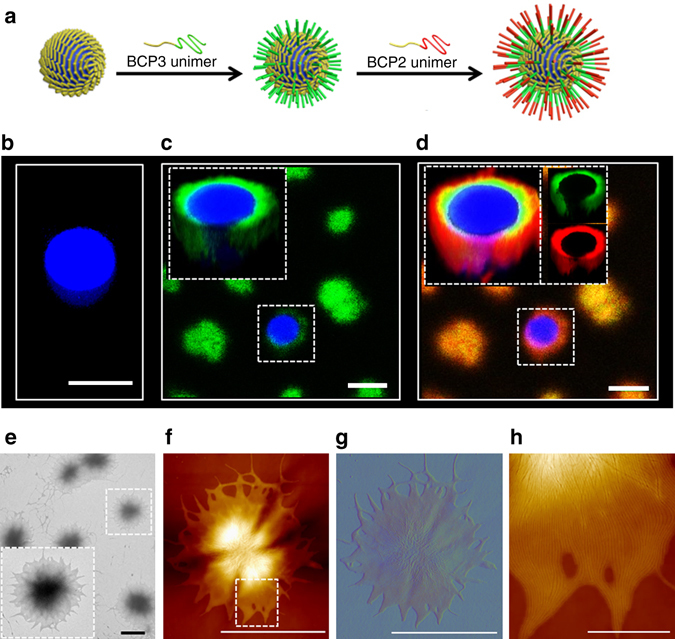
Membrane engineering of PFS_25_-*b*-PMVSCOOH_245_ colloidosomes by epitaxially driven micelle elongation. **a** Schematic representation showing structural and compositional elaboration of crosslinked BCP1 colloidosomes by a two-step sequence of epitaxial attachment of BCP1/BCP3 (*green*) and BCP1/BCP2 (*red*) unimers at the membrane surface. **b**–**d** Confocal fluorescence microscopy images of crosslinked colloidosomes prepared by interfacial assembly of Cascade blue–dextran-containing size-specific (*L*_n_ = 882 nm, *L*_w_/*L*_n_ = 1.01) BCP1 cylindrical micelles, and recorded before (**b**) and after addition of BCP1/BCP3 unimers (**c**), and after addition of BCP1/BCP2 unimers to the BCP1/BCP3-treated microcapsules (**d**). Epitaxial elongation of the BCP1 cylindrical micelles in the native membrane gives rise to successive shells of *green* and *red* fluorescence around the *blue* fluorescent interior of the colloidosomes. *Insets* show *Z*-stack images. Samples were dispersed in isopropanol at a colloidosome concentration of 0.1 mg mL^−1^ and unimer/colloidosome mass ratio of 7:5. Under these conditions, the dextran (*M*_w_ = 10,000 Da) was retained within the interior of the colloidosomes. In **c** and **d** the colloidosomes exhibit different colours in the low-magnification images as a result of their position relative to the confocal plane. **e** Representative TEM image showing hair-like texture on partially collapsed BCP1 colloidosomes after stepwise addition of BCP1/BCP3 and BCP1/BCP2 unimers; *inset* shows high-magnification view. **f**, **g** PeakForce AFM topography **f** and corresponding error signal (emphasises the edges of individual micelles) **g** of partially collapsed hairy colloidosome prepared as above. **h** High-resolution AFM topography of area delineated by the *dashed box* in **f** showing epitaxially elongated cylindrical micelles within the modified colloidosome membrane. The micelles are co-aligned and closely packed, and the length of the growth extension is between 1 and 2 μm. Samples were prepared at a unimer:colloidosome mass ratio of 7:5. *Scale bars* in **b**–**g**, 5 µm. *Scale bar* in **h**, 1 µm

The PFS_25_-*b*-PMVSCOOH_245_ colloidosome membrane could be chemically and structurally modified in the presence of BCP unimers by crystallization-driven epitaxial elongation of the PFS core domains exposed at the ends of the closely packed crosslinked cylindrical micelles. Specifically, we exploited the active growth behaviour of the micelle termini to generate a membrane consisting of three concentric shells that were structurally contiguous but exhibited different optical properties (Fig. 3a). For this, we prepared Cascade blue-labelled dextran-containing colloidosomes in EHOH using non-fluorescent BCP1 building blocks (*L*_n_ = 882 nm, *L*_w_/*L*_n_ = 1.01), transferred the crosslinked microstructures into isopropanol, added a tetrahydrofuran (THF) solution of BCP1/BCP3 (20 mg mL^−1^; 12.5 wt% BCP3; unimer:colloidosome mass ratio = 7:5; green fluorescence) and left the samples unstirred for 24 h, after which a solution of BCP1/BCP2 (20 mg mL^−1^; 12.5 wt% BCP2; unimer:colloidosome mass ratio = 7:5; red fluorescence) unimers was added and the colloidosomes left for a further 24 h. CLSM images revealed that the non-fluorescent BCP1 colloidosome membrane was encased in two distinct ca. 1 μm-thick concentric shells of green or red fluorescence that were adjacent to the native membrane or on the periphery of the modified microcapsules, respectively (Fig. 3b–d). Typically, growth of the shells occurred within 20 min (Supplementary Fig. 16). The spatial positioning of the green and red layers was consistent with the sequence of added fluorescent BCPs, suggesting that stepwise epitaxial elongation at the ends of the crosslinked BCP1 cylindrical micelle building blocks was responsible for post-assembly structural and compositional changes in the colloidosome membrane. This growth mechanism was confirmed by control experiments in which size-specific green fluorescent cylindrical micelles of BCP1/BCP3 (*L*_n_ = 789 nm, *L*_w_/*L*_n_ = 1.02) were added in place of a solution of BCP1/BCP3 unimers to a dispersion of crosslinked BCP1 colloidosomes in isopropanol. Under these conditions, the colloidosomes and preformed micelles remained physically separated and no changes in membrane texture were observed (Supplementary Fig. 17).

Partial collapse of the modified colloidosomes in the TEM and AFM revealed an elaborate hair-like membrane of closely packed cylindrical micelles (Fig. 3e–h). The constituent micelles were ca. 2 μm in length and co-aligned in the partially collapsed colloidosomes (Supplementary Fig. 18). Significantly, the lengths of the micelles measured by TEM and AFM were consistent with the total width of the green/red shell imaged by CLSM, suggesting that unlike the BCP1 cylinders of the native membrane, the hair-like extensions were oriented away from the membrane surface. Moreover, at a unimer:colloidosome mass ratio of 7:5, the increased length of the epitaxial outgrowths was equivalent to an approximate doubling in size of the native BCP1 cylindrical micelles. Further increases in the length of the hair-like outgrowths was achieved by increasing the number of added unimers and raising the unimer:colloidosome mass ratio to a value of 7:1 (Supplementary Fig. 19).

### Biofunctionalization of crystalline colloidosomes

**Fig. 4:**
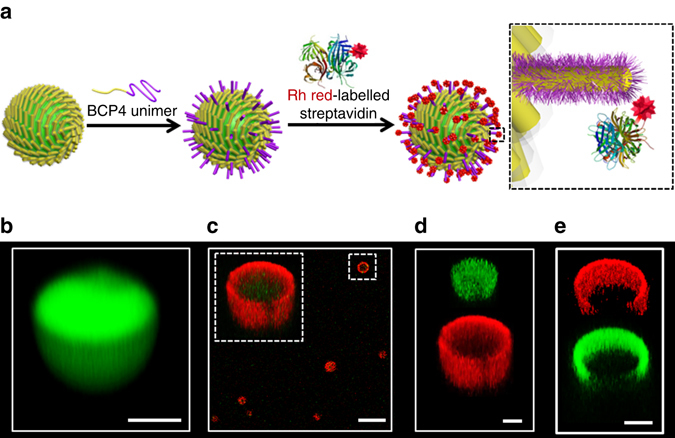
Biofunctionalization of PFS_25_-*b*-PMVSCOOH_245_colloidosomes. **a** Schematic representation showing preparation of biotinylated colloidosomes and membrane binding of streptavidin. Epitaxial attachment of BCP1/BCP4 unimers to the termini of BCP1 cylindrical micelles in the native membrane produces hair-like extensions with biotinylated side chains that serve as sites for streptavidin binding to produce biofunctionalized colloidosomes. **b**–**d** Confocal fluorescence microscopy images of crosslinked colloidosomes prepared by interfacial assembly of FITC–dextran-containing size-specific (*L*_n_ = 882 nm, *L*_w_/*L*_n_ = 1.01) BCP1 cylindrical micelles; **b** image showing *green* fluorescent interior in the native colloidosome confirming presence of encapsulated FITC–dextran (*M*_w_ = 70,000); **c**, **d** after addition of BCP1/BCP4 (1:1 weight ratio) unimers and subsequent conjugation of rhodamine-labelled streptavidin. *Z*-stack micrographs of individual colloidosomes based on merged *green*/*red* channel image (*inset* of **c**), and *green* or *red* channel images (**d**) confirm biotin-mediated binding of streptavidin to the epitaxially extended membrane of the colloidosomes. Samples were prepared in water/isopropanol mixtures at a volume ratio of 4:1. **e** Conjugation of biotinylated fluorescein–dextran to the hair-like membrane extensions of streptavidin-decorated biotinylated BCP1 water-filled colloidosomes; *Z*-stack micrographs of individual colloidosomes recorded in *red* or *green* channels confirming binding of biotinylated fluorescein–dextran to the rhodamine red–streptavidin functionalized colloidosomes. Samples were prepared in water/isopropanol mixtures at a volume ratio of 4:1. *Scale bars* in **b**, **d** and **e**, 2 µm. *Scale bar* in **c**, 10 µm

We used the above procedure to prepare biofunctionalized colloidosomes decorated with an array of biotinylated side chains that served as non-covalent binding sites for streptavidin (Fig. 4a). To achieve this we epitaxially attached a mixture of BCP1/BCP4 unimers to the termini of cylindrical micelles present in the membrane of crosslinked BCP1 colloidosomes to produce hair-like outgrowths displaying biotinylated side chains. Addition of rhodamine-tagged streptavidin resulted in the formation of a distinct outer shell of red fluorescence that was consistent with site-specific protein binding (Fig. 4b–d). Similar results were obtained for binding of FITC-streptavidin to the biotinylated colloidosomes (Supplementary Fig. 20). Control experiments in which unmodified crosslinked BCP1 colloidosomes were mixed directly with FITC-streptavidin showed no fluorescence associated with the diblock copolymer microcapsules in the absence of biotinylated side groups (Supplementary Fig. 21), indicating that non-specific binding was negligible under these conditions.

To determine whether the streptavidin-conjugated colloidosomes could be exploited as a potential biofunctionalized platform, we added biotinylated fluorescein–dextran to an aqueous suspension of the rhodamine-tagged streptavidin-decorated microcapsules. Confocal fluorescence images showed distinct red and green fluorescent outer shells corresponding to layers of conjugated streptavidin and dextran, respectively (Fig. 4e), consistent with non-covalent conjugation of the polysaccharide to free biotin-binding sites associated with the multivalency of the streptavidin tetramer. These proof-of-principle observations suggest that it should be possible to develop the hair-like biotinylated extensions of the colloidosome membrane as a high surface area platform for the streptavidin-mediated linkage of a wide range of antibodies and aptamers for use in areas such as diagnostics and therapeutics.

## Discussion

In summary, a PFS_25_-*b*-PMVSCOOH_245_ diblock copolymer with appropriate wettability and chemical reactivity was prepared and used to produce cylindrical core-shell nanostructures that serve as size-specific functional building blocks for the assembly and crosslinking of water-in-oil Pickering emulsion droplets. By encoding the diblock copolymer with structurally and chemically active domains associated with a short crystallizable PFS and long disordered PMVSCOOH block, respectively, colloidosomes in the form of semipermeable polymer microcapsules exhibiting unusual membrane structures and growth properties were produced. In particular, the colloidosomes can be chemically and structurally elaborated post assembly by in situ epitaxial elongation of the membrane-building blocks in the presence of unimers comprising fluorescent or biotinylated moieties. As a consequence, the colloidosome membrane transforms from a relatively smooth surface of closely packed cylindrical micelles oriented parallel to the shell surface into a dense network of hair-like outgrowths that are chemically distinct but contiguous with the native building blocks. We anticipate that the spatial and chemical extension introduced by post-assembly functionalization will offer increased opportunities for biomolecule capture and signalling compared with strategies involving functionalization of the rod-shaped micelles prior to colloidosome assembly.

The ability to prepare colloidosomes from structurally and chemically extendable building units that remain active post assembly could open up new avenues to customizable microcapsules for diverse applications such as the storage and delivery of functional molecules, spatially controlled micro-reactor catalysis, biomolecule conjugation and sensing, and design and construction of synthetic protocells^1, 7–19^. In this regard, it should be possible to decrease the colloidosome polydispersity by using appropriate microfluidic techniques^45^.Moreover, seed-mediated CDSA has also been used to prepare cylindrical block copolymer micelles of controlled length comprising crystalline cores of *π*-conjugated^46^, biodegradable^47^ and bioinert blocks^48^, suggesting that semiconducting and biocompatible colloidosomes could be fabricated using the above procedures. Finally, as the stability and permeability of polymer-based microcapsules are often influenced by the extent and reversibility of crosslinking^1, 49^, it should be possible to couple the epitaxial growth properties of PFS_25_-*b*-PMVSCOOH_245_colloidosome membranes with chemically controlled partial disassembly of intermicellar crosslinks to produce microcapsules capable of stimuli-responsive growth and reconfiguration.

## Methods

### Synthesis of BCPs BCP1–BCP4

Anionic polymerizations were performed in an argon-atmosphere glovebox. The PFS_25_-*b*-PMVS_245_ (BCP1, *M*_n_ = 27,250 g mol^−1^, PDI = 1.18; Supplementary Fig. 1; the degree of polymerization and block ratio were determined by gel permeation chromatography and ^1^H nuclear magnetic resonance (^1^H NMR) analyses) was prepared by previously reported methods^38, 39^. THF was distilled from Na/benzophenone immediately before use. Thiol-ene photoirradiation experiments were performed at 20 °C with Pyrex glass-filtered emission from a 125 W medium-pressure mercury lamp (Photochemical Reactors Ltd). In order to synthesize BCP1, 3-mercaptopropionic acid (661 mg, 0.54 mL, 6.24 mmol, ~ 10 eq.) and the photoinitiator DMPA (2.5 mg, 0.01 mmol) were added to a solution of PFS_25_-*b*-PMVS_245_(100 mg, 0.62 mmol vinyl) in dry THF (2 mL). The orange solution was sealed under a nitrogen atmosphere and irradiated 3 cm away from the mercury lamp for 2 h. The mixture was precipitated five times from hexane and dried in vacuo to afford the pure BCP1 (162 mg, 85%) as an orange adhesive solid. ^1^H NMR (400 MHz, pyridine-*d*_5_): *δ* 4.42 (s, 100 H, Cp*H*); 4.25 (s, 100 H, Cp*H*); 3.24–3.20 (m, 490 H, C*H*_2_COOH); 3.06–3.00 (m, 980 H, C*H*_2_SC*H*_2_); 1.35–1.32 (m, 490 H, SCH_2_C*H*_2_); 0.64 (s, 150 H, FcSi(C*H*_3_)_2_); and 0.48 (s, 735 H, SiC*H*_3_) p.p.m.

In some experiments (see synthesis of BCP2, BCP3 and BCP4 on pages 4–5 of the Supplementary Information), around 5% of the carboxylic acid groups in BCP1 were functionalized with different BODIPY fluorophores (BCP2, *red*; BCP3, *green*) or 10% with biotin side groups (BCP4).

### Preparation of cylindrical micelles

To prepare polydisperse cylindrical micelles (*L*_n_ = 3870 nm, *L*_w_/*L*_n_ = 1.23), a screw-cap vial containing 3 mg of BCP1 in solid and 6 mL of EHOH was sealed and heated to 70 °C with stirring. After 1 h, the stirring and heating was stopped and the clear yellow solution left to cool to room temperature and aged for 72 h. The solution of the polydisperse cylindrical micelles as above was then cooled to 0 °C and sonicated for 1 h to afford a solution of short BCP seed micelles (*L*_n_ = 43 nm, *L*_w_/*L*_n_ = 1.05). To prepare size-specific cylindrical micelles with specific lengths, a designated amount of BCP1 (for the preparation of fluorescent cylindrical micelles a mixture of BCP1 and fluorescent BCP2 or 3 was used; for details please see preparation of BCP2 and BCP3 fluorescent micelles in page 9 of Supplementary Information) unimer solution in THF (20 mg mL^−1^) was rapidly added dropwise to a stirred (500 r.p.m.) 0.5 mL solution of freshly prepared BCP1 seed micelles in EHOH or isopropanol (iPrOH) at a designated concentration. After 5 s, the stirring was stopped and the solutions were aged for 24 h.

### Preparation of colloidosomes

Colloidosomes were prepared by mixing a designated amount of Milli-Q water with the oil phase, the latter of which was an EHOH solution in which the BCP cylindrical micelles were dispersed at a designed concentration. A BCP micelle-stabilized Pickering emulsion was then formed by shaking the mixture at a speed of 3000 r.p.m. using a vortex mixer. The samples were prepared at a designated water/oil volume fraction (*φ*_w_) that ranged between 0.03 and 0.30, and at a BCP concentration that ranged between 0.25 and 4.0 mg mL^−1^. Typically, 0.025 mL of water was mixed with 0.500 mL of the BCP solution in EHOH. Colloidosomes containing encapsulated components, including dye-labelled dextrans (FITC–dextran, Cascade blue-conjugated dextran) were prepared according to the above procedures except that the encapsulants were added directly to the inner water phase before it was mixed with the oil phase. Crosslinked colloidosomes were prepared by adding crosslinker directly into the water phase (see preparation of crosslinked colloidosomes in page 9 of Supplementary Information).

### Membrane engineering of BCP1 colloidosomes

Epitaxially mediated membrane extension was performed from the BCP1-based crosslinked colloidosomes in which dye-labelled dextrans were encapsulated. Typically, to 500 μL of a dispersion of crosslinked colloidosomes prepared from 882 nm-long BCP1-based cylindrical micelles in iPrOH (0.1 mg mL^−1^) was added 3.5 μL of a BCP2/BCP1 mixed unimer solution in THF (*W*_BCP2_/*W*_BCP1_ = 1/7, *C* = 20 mg mL^−1^). This mixture was shaken immediately using a vortex mixer at 1000 r.p.m. After 10 s, the stirring was stopped and the solution was aged for 24 h to allow growth of the green fluorescent hair-like shell. Subsequently, 3.5 μL of a BCP3/BCP1 mixed unimer solution in THF (*W*_BCP3_/*W*_BCP1_ = 1/7, *C* = 20 mg mL^−1^) was added according to the same procedure to allow the contiguous growth of a red fluorescent hair-like shell. In order to prepare the crosslinked colloidosomes with longer membrane outgrowths, 17.5 μL of BCP unimer solutions were added to facilitate the growth of both the green and red shells. To determine the duration required for the micelle elongation from the colloidosome membrane and to confirm the epitaxial elongation of the membrane-building blocks, a kinetic study and a blank experiment were performed (see membrane engineering of BCP1 colloidosomes—kinetic study and control experiment on page 9 of Supplementary Information).

### Micelle and colloidosome characterization

The characterization by using TEM, AFM, CLSM, OM and fluorescence microscopy is described in pages 5–7 of Supplementary Information.

### Data availability

The data that support the findings of this study are available from the corresponding authors upon reasonable request.

## Electronic supplementary material

Supplementary Information
