# Supplementary material for: Higher-order assembly of crystalline cylindrical micelles into membrane-extendable colloidosomes
Source: Nat Commun. 2017 Sep 4;8:426. doi: 10.1038/s41467-017-00465-z (PMC5583177; doi:10.1038/s41467-017-00465-z)
Supplement: Supplementary file 1 — Supplementary Information [file 41467_2017_465_MOESM1_ESM.pdf]

Revised NCOMM -17-03015C

Supplementary Information for

Higher-order assembly of crystalline cylindrical micelles into membrane-extendable colloidosomes

Hongjing Dou[1, 2], Mei Li[1], Yan Qiao[1†], Robert Harniman[1†], Xiaoyu Li[1], Charlotte E. Boott[1], Stephen Mann[1*] and Ian Manners[1*]

* To whom correspondence should be addressed: ian.manners@bristol.ac.uk (I.M.), s.mann@bristol.ac.uk (S.M.)

† These authors contributed equally to this work.

This file includes:

List of Acronyms and Abbreviations

Supplementary Methods

Figs. S1 to S21

Full Reference List

Supplementary Methods

Materials. 3-Mercaptopropionic acid (Sigma-Aldrich, ≥99%), the photoinitiator 2,2-dimethoxy-2-phenylacetophenone (DMPA), 5-(((4-(4,4-difluoro-5-(2-thienyl)-4-bora-3a,4a-diaza-*s*-indacene-3-yl)phenoxy)acetyl)amino)pentylamine, hydrochloride (BODIPY TR cadaverine, Thermo Fisher Scientific), 4,4-difluoro-5,7-dimethyl-4-bora-3a,4a-diaza-*s*-indacene-3-propionic acid, hydrazide (BODIPY FL hydrazide, Thermo Fisher Scientific), EZ-link™ hydrazide-biotin (Thermo Fisher Scientific), 1-[3-(dimethylamino)propyl]-3-ethylcarbodiimide hydrochloride (EDC-HCl, Alfa Aesar), 1-hydroxybenzotriazole hydrate (HBT, Aldrich, ≥97%), 1-[3-(dimethylamino)propyl]-3-ethylcarbodiimide methiodide (EDCI, Aldrich), *N*-hydroxysuccinimide (NHS, Fluka, > 97%), 2,2'-(ethylenedioxy)bis(ethylamine) (EDEA, Aldrich, 98%), and 2-ethyl-1-hexanol (Aldrich, 98%), were used as received without further purification. Additionally, a series of four fluorescein isothiocyanate-dextran (FITC-dextran) polymers with various molecular weights (M_w = 4,400, 10,000, 70,000, and 150,000 g·mol^{-1}, respectively, Sigma), as well as dextran-Cascade Blue (dextran-CB) (M_w = 10,000 g·mol^{-1}, anionic, lysine fixable, Thermo Fisher Scientific), streptavidin-fluorescein isothiocyanate (FITC) conjugate (Thermo Fisher Scientific), streptavidin-Rhodamine (Rh) red conjugate (Thermo Fisher Scientific), and biotinylated fluorescein-dextran (Dextran, Fluorescein and Biotin, 10,000 MW, Anionic, Lysine Fixable) were used as received without further purification.

Polymer Characterization. ^{1}H nuclear magnetic resonance (NMR) spectra were recorded using a Varian VNMR 400 MHz spectrometer. MALDI-TOF mass spectrometry measurements of the poly(ferrocenyldimethylsilane) (PFS) homopolymer were performed using a Bruker Ultraflextreme system that was operated in the linear mode. Samples were prepared by mixing a *trans*-2-[3-(4-*tert*-butylphenyl)-2-methyl-2-propenylidene]malononitrile matrix (20 mg·mL^{-1} in tetrahydrofuran, THF) with the polymer sample (2 mg·mL^{-1} in THF) at a 10:1 (v/v) ratio. The polydispersity index (PDI = M_w/M_n) of BCP 1 was determined by Gel Permeation Chromatography (GPC) using THF as eluent phase with a Viscotek VE 2001 Triple-Detector Gel Permeation Chromatograph System that was equipped with an automatic sampler, a pump, an injector, an inline degasser, a column oven (30 °C), styrene/divinylbenzene columns (pore sizes of between 500 Å and 100,000 Å), a VE 3580 refractometer, a four-capillary differential

viscometer, a VE 3210 UV/Vis detector (λ = 450 nm) and a VE 270 dual angle laser light scattering detector (7° and 90°). Calibration of the detectors was performed using polystyrene standards (Viscotek). The carboxyl group bearing BCPs 2-4 were not characterized by GPC due to possible interactions with the GPC column.

Synthesis of PFS_{25}-b-($PMVSCOOH_x$-r-G_{1-x})$_{245}$ (BCP 2). BCP 1 (20.0 mg, bearing 0.100 mmol of carboxyl groups) was dissolved in 0.30 mL of anhydrous dimethylformamide. To this solution, under vigorous stirring and nitrogen bubbling, was then added 10.0 mg (0.0300 mmol) of EDC-HCl and 6.9 mg (0.045 mmol) of HBT dissolved in 0.30 mL of anhydrous dimethylformamide. After 15 min, 1.5 mg (0.0050 mmol, ~0.05 eq.) of BODIPY FL hydrazide in 0.05 mL of anhydrous dimethylformamide was added. 8 h later, another 10.0 mg (0.0300 mmol) of EDC-HCl and 6.9 mg (0.045 mmol) of HBT in 0.10 mL of anhydrous dimethylformamide were added into the mixture. The reaction was stirred under an argon atmosphere at room temperature for another 24 h. The mixture was dialyzed (dialysis tubing 12–14 kDa MWCO) against Milli-Q water and then THF to remove any excess catalyst before it was subsequently precipitated three times into hexane to afford 18 mg (yield 80%) of an orange adhesive solid. ^{1}H NMR analysis indicated that 5% of the carboxyl functional groups had been functionalized with the BODIPY FL dye, while 95% of the carboxyl groups remained in their pristine form. ^{1}H NMR (400 MHz, THF-d_4): δ 7.38 (s, 12H, Ar*H*), 6.94 (s, 12H, Ar*H*), 6.38 (s, 12H, Ar*H*), 6.15 (s, 12H, Ar*H*), 4.22 (s, 100H, Cp*H*), 4.05 (s, 100H, Cp*H*), 2.83-2.75 (m, 490H of $CH_2C(O)$ and 48H of $NHC(O)CH_2CH_2$), 2.71-2.50 (m, 980H, CH_2SCH_2), 1.06-0.89 (m, 490H, SCH_2CH_2), 0.48 (s, 150H, $FcSi(CH_3)_2$), 0.24-0.11 (m, 735H, $SiCH_3$) ppm.

Synthesis of PFS_{25}-b-($PMVSCOOH_x$-r-R_{1-x})$_{245}$ (BCP 3). The synthetic process employed to prepare BCP 3 was similar to that used to prepare BCP 2, except that 2.7 mg (0.0050 mmol) of BODIPY TR cadaverine was used instead of 1.5 mg (0.0050 mmol, ~0.05 eq.) of BODIPY FL hydrazide. After the reaction, the mixture was dialyzed (dialysis tubing 12–14 kDa MWCO) against Milli-Q water and then THF to remove any excess catalyst before the solution was precipitated three times from hexane to afford 19 mg (yield 77%) of a dark purple adhesive solid. ^{1}H NMR analysis indicated that 5% of the carboxyl functional groups had been functionalized with BODIPY TR cadaverine dye, while 95% of the carboxyl groups remained in their pristine

form. ^{1}H NMR (400 MHz, THF-d_4): 8.02 (d, 12H, thienyl Ar-H), 7.60 (d, 24H, -C_6H_4-), 7.44 (d, 12H, thienyl Ar-H), 7.21 (t, 12H, thienyl Ar-H), 7.15 (s, 12H, C*H* between pyrroles), 7.12 (d, 12H, pyrrole Ar-H), 7.08 (d, 12H, pyrrole Ar-H) 7.03 (d, 12H, pyrrole Ar-H), 6.88 (d, 24H, -C_6H_4-), 6.79 (d, 12H, pyrrole Ar-H), 4.39 (t, 24H, CH_2OR), 4.22 (s, 100H, Cp*H*), 4.05 (s, 100H, Cp*H*), 3.75 (m, 24H, $NCH_2CH_2CH_2CH_2CH_2N$), 3.39 (m, 24H, $NCH_2CH_2CH_2CH_2CH_2N$), 2.83-2.75 (m, 490H of CH_2C(O) and 24H of NHC(O)CH_2), 2.71-2.50 (m, 980H, CH_2SCH_2), 1.89 (m, 24H, $NCH_2CH_2CH_2CH_2CH_2N$), 1.57 (m, 24H, $NCH_2CH_2CH_2CH_2CH_2N$), 1.06-0.89 (m, 490H, SCH_2CH_2), 0.88 (m, 24H, $NCH_2CH_2CH_2CH_2CH_2N$), 0.48 (s, 150H, $FcSi(CH_3)_2$), 0.24-0.11 (m, 735H, $SiCH_3$) ppm.

Synthesis of PFS_{25}-b-$(PMVSCOOH_x$-r-$Biotin_{1-x})_{245}$ (BCP 4). BCP 1 (15.0 mg, 0.0750 mmol of carboxyl groups) was dissolved in 0.20 mL of anhydrous dimethylformamide. To this solution, under vigorous stirring and nitrogen bubbling, was then added 7.5 mg (0.024 mmol) of EDC-HCl and 5.3 mg (0.033 mmol) of HBT dissolved in 0.10 mL of anhydrous dimethylformamide. After 15 min, 1.9 mg (0.0070 mmol, ~0.10 eq.) of EZ-link™ hydrazine-biotin in 0.05 mL of anhydrous dimethylformamide was added to this mixture. Another 7.5 mg (0.024 mmol) of EDC-HCl and 5.3 mg (0.033 mmol) of HBT in 0.30 mL of anhydrous dimethylformamide were added into the mixture 15 h later. The reaction was stirred under an argon atmosphere at room temperature for another 24 h. The mixture was dialyzed (with dialysis tubing having a MWCO of 12–14 kDa) against Milli-Q water and then THF to remove any excess catalyst. Subsequently, the product was precipitated three times via addition into hexane to afford 12 mg (yield 67%) of an orange solid. ^{1}H NMR analysis indicated that 10% of the carboxyl functional groups had been functionalized with biotin, while 90% of the carboxyl groups remained in their pristine form. Here the ratio of biotinylated carboxylic groups was calculated by comparing the integral peak area of the NHC*H* in the ketone of the imidazole ring (δ 4.50) and the Cp*H* (δ 4.43). ^{1}H NMR (400 MHz, d_5-pyridine): δ 4.50 (s, 24H, NHC*H* in the ketone of the imidazole ring), 4.43 (s, 100H, Cp*H*), 4.31 (s, 24H, NHC*H* in the ketone of the imidazole ring), 4.25 (s, 100H, Cp*H*), 3.24-3.20 (m, 490H, CH_2COOH), 3.04-3.00 (m, 980H, CH_2SCH_2), 2.88-2.80 (m, 72H, $CHSCH_2$ in the thienyl ring), 2.44 (s, 48H, C(O)CH_2), 1.88-1.73 (m, 144H, C(O)$CH_2CH_2CH_2CH_2$), 1.35-1.32 (m, 490H, SCH_2CH_2), 0.64 (s, 150H, $FcSi(CH_3)_2$), 0.48 (s, 735H, $SiCH_3$) ppm.

Transmission electron microscopy (TEM). TEM analysis was performed with a Jeol 1400 TEM using a LaB6 filament at 140 kV. The samples were prepared by drop-casting one drop (*ca.* 5 μL) of solution onto a carbon coated copper grid, and the specimens were then dried in the ambient atmosphere for 24 h. Images were analyzed using the ImageJ software package developed at the US National Institute of Health. The lengths of the BCP micelles and their size distributions were determined by tracing 200 positions. Taking the micelle structure as an example, the number average lengths (L_n) and weight average lengths (L_w) of the micelles were calculated as shown in supplementary Equation 1 and 2:

$$L_n = \frac{\sum_{i=1}^{N} N_i L_i}{\sum_{i=1}^{N} N_i} \quad (1)$$

$$L_W = \frac{\sum_{i=1}^{N} N_i L_i^2}{\sum_{i=1}^{N} N_i L_i} \quad (2)$$

where N_i is the number of micelles of length L_i, and N is the total number of micelles examined for each sample. The distribution of micelle lengths is characterized by L_w/L_n.

Atomic force microscopy (AFM). AFM topographic images were recorded using a Multimode VIII atomic force microscope with a Nanoscope V controller utilizing PeakForce feedback (Bruker, CA, USA). Samples were prepared drop casting 5 μL of solution onto a carbon film coated finder grid (Agar Scientific). Likely candidates for AFM investigation were then identified via TEM imaging before the finder grid was mounted within the AFM system. Imaging was conducted in an ambient environment using a fast scan ScanAsyst-HR head unit. A fresh SCANASYST-HR cantilever, with a nominal tip radius of 2nm, (Bruker, CA, USA) was used for each different sample. Quantitative nano-mechanical mapping (QNM) of the sample's Young's Modulus was conducted using an RTESPA cantilever (Bruker, CA, USA). The detection sensitivity of the system was calibrated using the relative method and a calibration sample of PS-film (Bruker, CA, USA) with a nominal Young's modulus of 2.7 GPa. A sample of BCP 1-based colloidosome dispersed in EHOH was drop cast onto freshly cleaved muscovite mica and air dried overnight before being investigated in an ambient environment.

Confocal laser scanning microscopy (CLSM). CLSM images were obtained using a Leica SP5 system, which was attached to a Leica DM I6000 inverted epifluorescence microscope with a 63× oil immersion objective lenses with a numerical aperture (NA) = 1.4. Fluorophores from BCP 2 and Rh red-streptavidin were excited using a HeNe laser that was operated at 594 nm, while fluorophores from BCP 3, FITC-dextran, FITC-streptavidin, and biotinylated fluorescein-dextran were excited using an argon laser operating at 488 nm, and fluorophores from cascade blue-labeled dextran were excited using a 50 mW 405 nm diode laser. Confocal images were obtained using digital detectors with observation windows of 650 - 710 nm for BCP 3, 640 - 700 nm for Rh red-streptavidin, 530 - 590 nm for BCP 2, 550 - 600 nm for FITC-dextran and biotinylated fluorescein-dextran, and 440 – 500 nm for cascade blue-labeled dextran. The resulting outputs were obtained as digital false-colour images, and colour coded as red, green, and blue, respectively. To prepare a sample for CLSM characterization, a rectangular capillary tube (0.10 mm × 1.00 mm, 50 mm length) was immersed in the sample solution until it was filled with the solution. The capillary tube was then laid down on an optical microscopy glass slide and the two ends were sealed with epoxy resin glue.

Optical microscopy and fluorescence microscopy (OM and FM). Optical and fluorescence microscopy (abbreviated as OM and FM, respectively) was performed using a Leica DMI3000 B manual inverted fluorescence microscope at 63x and 100x magnification. A fluorescence filter with excitation at 340-380 nm and an emission cut-off at 400 nm was used. The samples were prepared for OM and FM characterization in a similar manner as had been employed for CLSM characterization. In order to calculate the average size of the colloidosomes, OM images were analyzed using the ImageJ software package developed at the US National Institute of Health. The diameters of the colloidosomes and their size distributions were determined by measuring 200 objects. The number average diameters (D_{n}) and corresponding standard deviation (S) of the colloidosomes were calculated using supplementary equation 3 and 4, where N_{i} is the number of colloidosomes of diameter D_{i}, and N is the total number of colloidosomes examined for each sample.

$$D_{\mathrm{n}} = \frac{\sum_{i=1}^{N} N_i D_i}{\sum_{i=1}^{N} N_i} \qquad (3)$$

$$S=\sqrt{\frac{1}{N-1}\sum_{i=1}^{N}(D_i-D_n)^2} \quad (4)$$

Ultraviolet/visible (UV-vis) and fluorescence spectroscopy. UV-vis and fluorescence emission spectra of the solutions of the illuminated micelles in EHOH were recorded using a PerkinElmer Lambda 25 spectrophotometer and a Jasco FP-6500 fluorimeter, respectively.

Seeded growth of BCP unimers in EHOH and isopropanol (iPrOH) by using 43 nm (in EHOH, L_n = 43 nm, L_w/L_n = 1.05) and 41 nm (in iPrOH, L_n = 41 nm, L_w/L_n = 1.06) BCP 1 seed micelles. Seeded growth approach was used to prepare size-specific BCP cylindrical micelles [S1-S3]. In order to investigate the dependence of the micelle length on the unimer:seed ratio in the seeded growth of BCP 1, a designated amount BCP 1 unimer solution in THF (20 mg·mL^{-1}) was rapidly added dropwise to a stirred (500 rpm) 0.5 mL solution of freshly prepared BCP 1 seed micelles in EHOH or iPrOH (0.1 mg·mL^{-1}). After 5 s, the stirring was stopped and the solutions were aged for 24 h. The growth of the cylindrical micelles was monitored by TEM (Supplementary Figure 3 and 10) and the L_w and L_n values were respectively calculated according to supplementary Equations 1 and 2.

The 41 nm BCP1 seed micelles in iPrOH were prepared in the same manner as the procedure for preparing 43 nm BCP1 seed micelles in EHOH, except that iPrOH was used as solvent instead of the EHOH.

Preparation of 320, 882 and 1129 nm BCP 1 micelles. In order to prepare the 320 nm BCP 1 micelles (L_n = 320 nm, L_w/L_n = 1.06) and keep the final concentration of the micelles at 0.5 mg·mL^{-1} to facilitate the subsequent preparation of BCP 1 colloidosomes, 24 μL of a BCP 1 unimer solution in THF (20 mg·mL^{-1}) was rapidly added dropwise to a stirred (500 rpm) 1 mL solution of freshly prepared BCP 1 seed micelles in EHOH (0.029 mg·mL^{-1}). After 5 s, the stirring was stopped and the solutions were aged for 24 h. Subsequently, the solution was gently bubbled with nitrogen for 30 min to remove any trace amounts of THF that had been introduced with the addition of the unimer solution. The growth of the cylindrical micelles was monitored by TEM, while the L_w and L_n of the micelles were calculated according to Equations S1 and S2.

The 882 (L_n = 882 nm, L_w/L_n = 1.01), and 1129 nm BCP 1 micelles (L_n = 1129 nm, L_w/L_n =

1.02) were prepared in the same manner as above except that a more dilute BCP 1 seed micelle solution was used instead of the 0.029 $mg \cdot mL^{-1}$ seed micelle solution to keep the final BCP 1 concentration of 0.5 $mg \cdot mL^{-1}$.

Transfer of the well-defined cylinders to iPrOH and water. In order to confirm the narrow size dispersity of BCP 1 micelles in iPrOH and water, 882 nm (L_n = 882 nm, L_w / L_n = 1.01) BCP 1 micelles that had been prepared via seeded growth in EHOH (C = 0.5 $mg \cdot mL^{-1}$) were diluted via the addition of iPrOH to obtain a dispersion of these micelles in a iPrOH/EHOH mixture (V_{iPrOH}/V_{EHOH} = 4/1, C = 0.1 $mg \cdot mL^{-1}$). Subsequently, the solution was dialyzed against a series of water/iPrOH mixtures with a decreasing iPrOH volume fraction in order to convert the dispersion solvent to water. The micelles dispersed in iPrOH/EHOH mixtures or water were then observed by TEM.

Seeded growth of BCP 3 unimers from 43 nm (L_n = 43 nm, L_w/L_n = 1.05) BCP 1 seed micelles. For the investigation of the seeded growth of BCP 3 unimers from 43 nm BCP 1 seed micelles, the same procedure was employed as for the seeded growth investigation of BCP 1 unimers, except that a BCP 3/BCP 1 unimer mixture at a mass ratio of 1/7 (20 $mg \cdot mL^{-1}$) was used instead of the BCP 1 unimer solution. Similarly, the growth of the cylindrical micelles was monitored by TEM, while the L_w and L_n values were respectively calculated according to supplementary Equations 1 and 2.

Preparation of 756 nm BCP 2 and 789 nm BCP 3 fluorescent micelles. In order to prepare the 756 nm BCP 2 fluorescent micelles (L_n = 756 nm, L_w/L_n = 1.02) and keep the final concentration of the micelles at 0.5 $mg \cdot mL^{-1}$ (to thus facilitate the subsequent preparation of colloidosomes), 24 μL of a BCP 2/BCP 1 mixed unimer solution in THF (W_{BCP2} / W_{BCP1} = 1/7, C = 20 $mg \cdot mL^{-1}$) was rapidly added dropwise to a stirred (500 rpm) 1 mL solution of freshly prepared BCP 1 seed micelles in EHOH (0.012 $mg \cdot mL^{-1}$). After 5 s, the stirring was stopped and the solutions were aged for 24 h. Subsequently, the solution was gently bubbled with nitrogen for 30 min to remove any trace amounts of THF that had been introduced with the addition of the unimer solution. The growth of the cylindrical micelles was monitored by TEM, while the L_w and L_n values of the micelles were respectively calculated according to supplementary Equations S1 and S2.

The 789 nm BCP 3 micelles (L_n = 789 nm, L_w/L_n = 1.02) were prepared according to the same procedure as has been used to prepare the 756 nm BCP 2 micelles, expect that a BCP 3/BCP 1 mixed unimer solution in THF (W_{BCP3}/W_{BCP1} = 1/7, C = 20 mg·mL^{-1}) was used.

Preparation of crosslinked colloidosomes. Crosslinked colloidosomes were prepared by adding the crosslinker together with the catalyst in the water phase before it was mixed with the oil phase. Typically, 9.2 μL of an aqueous solution of EDEA (20 mg·mL^{-1}, *ca.* 2 *eq.* regarding carboxyl groups, adjusted to a pH of 6.5 using 5 M HCl), 9.2 μL of an aqueous solution of EDCI (40 mg·mL^{-1}), 9.2 μL of an aqueous solution of NHS (40 mg·mL^{-1}), and 22.4 μL of Milli-Q water were pre-mixed as a water phase. This water phase was subsequently mixed with 0.5 mL of an EHOH oil phase before it was agitated using a vortex mixer. The crosslinking reaction was allowed to proceed for 48 h. At the end of the crosslinking reaction, the colloidosomes settled to the bottom of the plastic vials, and the upper oil layer was discarded. After the addition of 0.45 mL of iPrOH into the vial, the solution was dialyzed against a series of water/iPrOH mixtures with increasing water volume fractions. By following this procedure, the colloidosomes were thus transferred into bulk water or iPrOH solutions. In order to prepare colloidosomes encapsulating dye-labeled biomolecules, a mixture of 17.4 μL of water and 5 μL of an aqueous biomolecule solution (5 mg·mL^{-1}) was mixed with the other reactants instead of 22.4 μL of water.

Membrane engineering of BCP 1 colloidosomes – kinetic study and control experiment. A kinetic study was performed to determine the duration required for the micelle elongation from the colloidosome membrane. To achieve this, the sample was observed via CLSM 5min, 20 min, 60 min and 120 min after mixing the unimer solution with a dispersion of cross-linked colloidosomes. The kinetic study confirmed that the growth of the hair-like extensions was a rapid process and was primarily accomplished within 20 min.

In order to confirm epitaxial elongation of the membrane building blocks, a blank experiment was performed. In particular, 200 μL of BCP 1-based cross-linked colloidosomes in iPrOH (0.1 mg·mL^{-1}) was mixed directly with 60 μL of an iPrOH solution of 789 nm BCP 3 fluorescent micelles (0.5 mg·mL^{-1}). This solution was aged for 24 h prior to the CLSM observation, which confirmed that no filament growth had occurred.

Preparation of the biotin-capped cross-linked colloidosomes and the subsequent capture of streptavidin and additional layer of biotinylated fluorescein-dextran. In order to prepare the biotin-capped cross-linked colloidosomes, 5 μL of a BCP 4/BCP 1 mixed unimer solution in THF (W_{BCP4}/W_{BCP1} = 1/1, C = 10 mg·mL^{-1}) was added to 100 μL of a BCP 1-based colloidosome (L_n = 882 nm) dispersion in iPrOH (0.5 mg·mL^{-1}). This mixture was immediately shaken using a vortex mixer at 1000 rpm. After 10 s, the stirring was stopped and the solution was aged for 24 h to allow the growth of the biotin-capped membrane outgrowths. Subsequently, a 420 μL aqueous solution of Rh red-streptavidin (5×10^{-3} mg·mL^{-1}) was added into the 105 μL of the biotinyated colloidosomes in iPrOH. This mixture was shaken at 1000 rpm for 5 s and was then aged in an ambient environment for 2 h prior to CLSM observation. In order to label the inner water phase, FITC-dextran- or dextran-CB-containing cross-linked colloidosomes were used in the preparation of the biotinylated colloidosomes.

In the study on the binding of additional layer of biotinylated fluorescein-dextran to the streptavidin-conjugated colloidosomes, we used water-filled BCP 1-based colloidosome (L_n = 882 nm) to prepare the biotin-capped cross-linked colloidosome. Subsequently, Rh red-streptavidin capture was achieved following same procedure as above. Thereafter, a 2.2 μL aqueous solution of biotinylated fluorescein (1 mg·mL^{-1}) was added into the 600 μL of the streptavidin-conjugated colloidosomes in iPrOH to facilitate the binding of biotinylated dextran macromolecules.

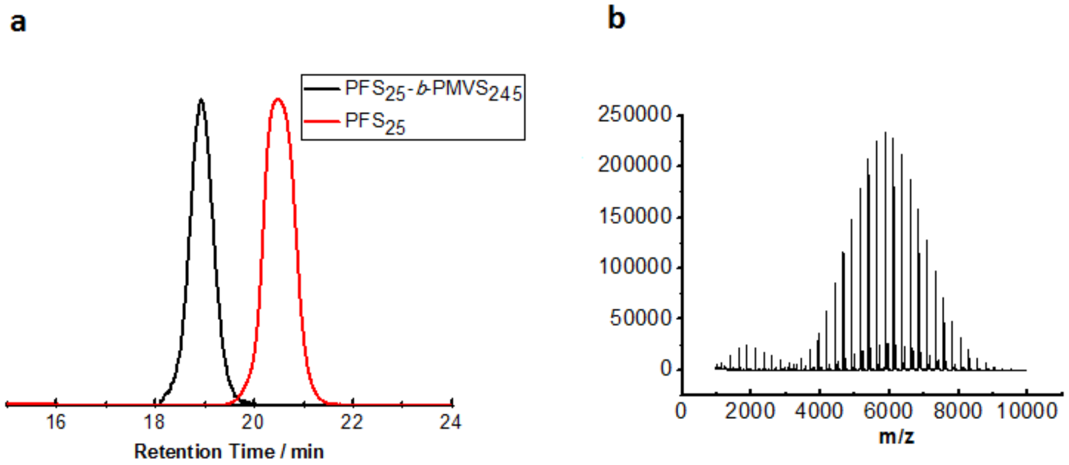

Supplementary Figure 1 | Superimposed GPC traces (RI versus retention time) (a) of PFS_{25} and PFS_{25}-*b*-$PMVS_{245}$ and MALDI-TOF mass spectrum of PFS_{25} (b). Tetrahydrofuran (THF) was used as the eluent. The PDI value of PFS_{25}-*b*-$PMVS_{245}$ was 1.18.

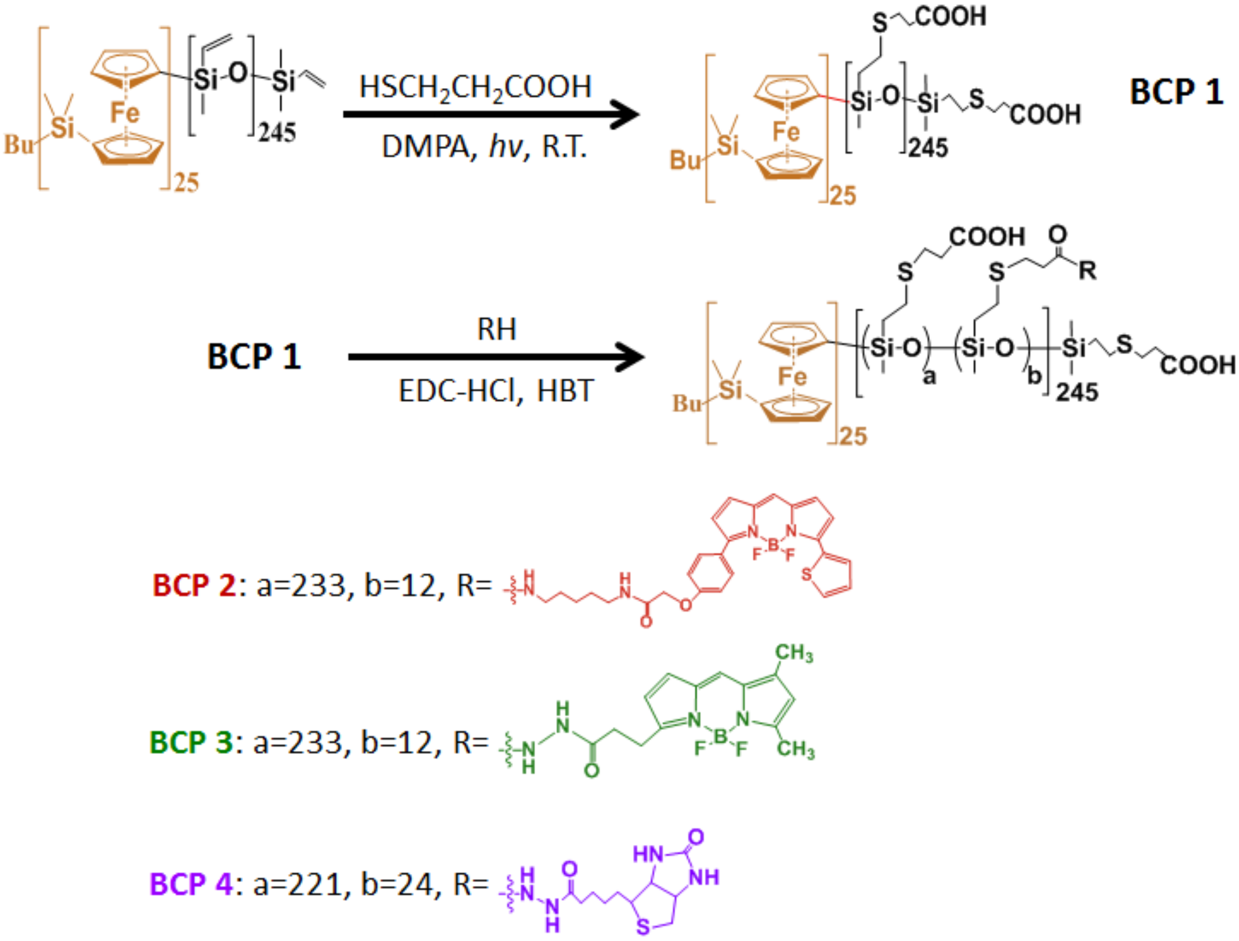

Supplementary Figure 2 | Synthesis of block copolymers BCP1-4. DMPA, EDC-HCl, and HBT denote 2,2-dimethoxy-2-phenylacetophenone, [3-(dimethylamino)propyl]-3-ethylcarbodiimide hydrochloride, and 1-hydroxybenzotriazole hydrate, respectively.

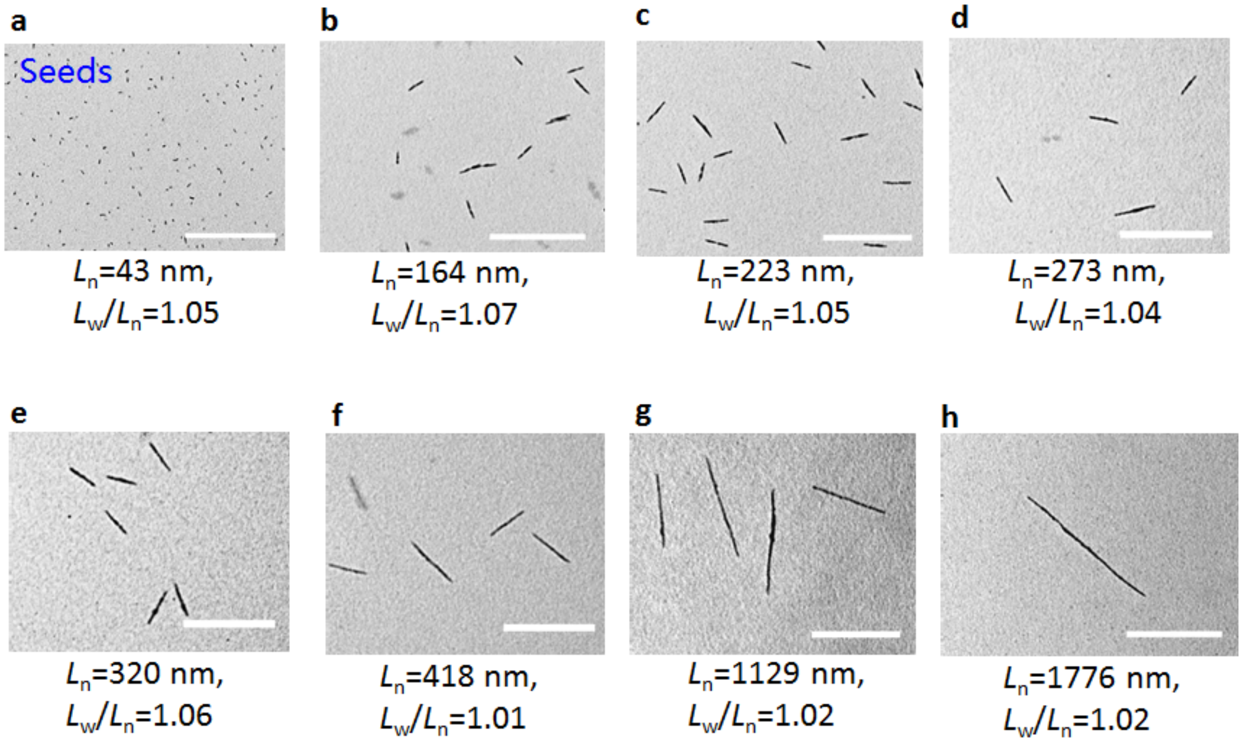

Supplementary Figure 3 | TEM images showing uniform cylindrical BCP1 micelles with controlled lengths prepared by seeded growth in EHOH. (**a**)-(**h**) Cylinder growth proceeds via the addition of unimers to the crystalline PFS core regions exposed at the tips of the micelle seeds. (**a**) BCP1 seeds. (**b-h**) growth at unimer : seed mass ratios of (**b**) 4:1, (**c**) 8:1, (**d**) 12:1, (**e**) 16:1, (**f**) 24:1, (**g**) 50:1 and (**h**) 100:1. Scale bars, 1000 nm.

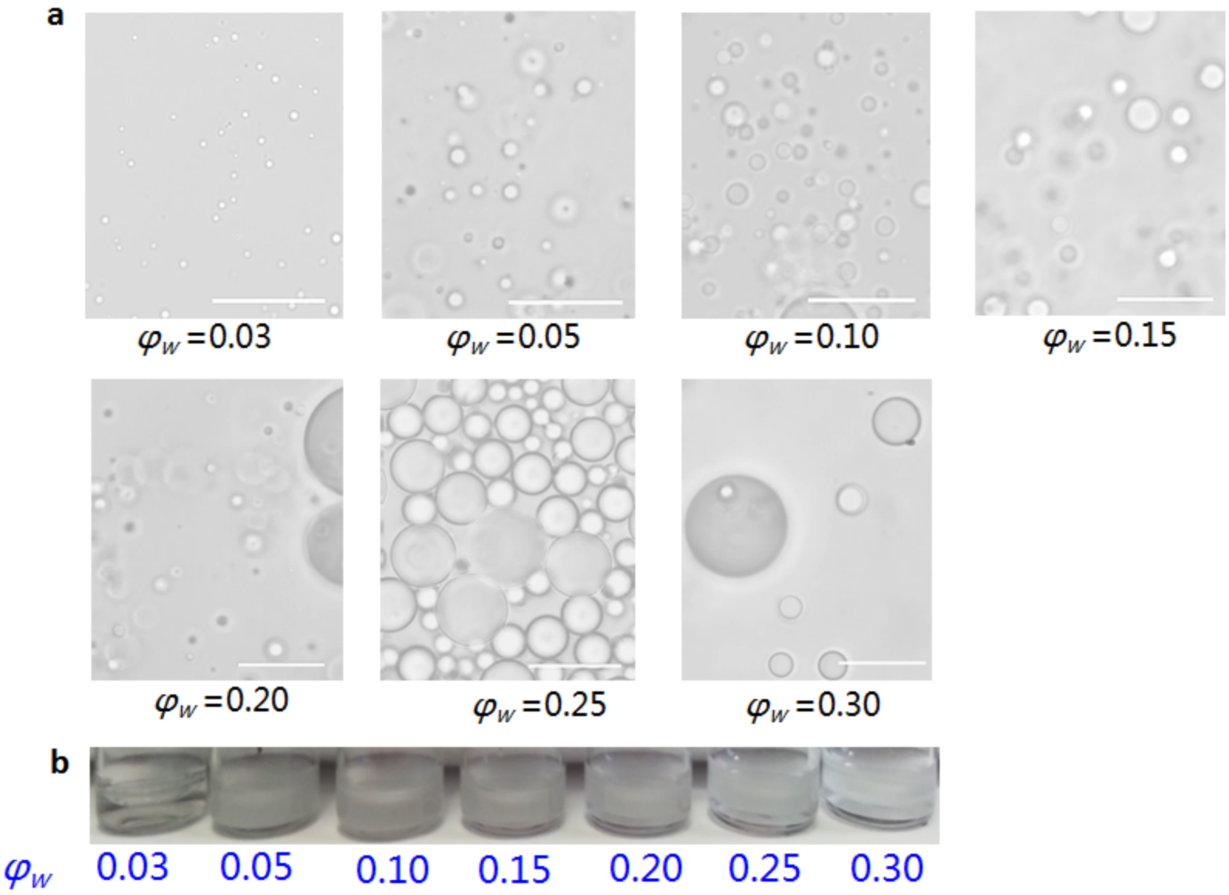

Supplementary Figure 4 | Optical microscopy images (a) and photographic images (b) of water-in-oil Pickering emulsions prepared from BCP1 cylindrical micelles (L_n = 43 nm) at different water/oil volume fractions (ϕ_w). The BCP1 concentration was kept constant at 0.5 mg mL^{-1} in the EHOH oil phase. Scale bars, 20 µm.

a

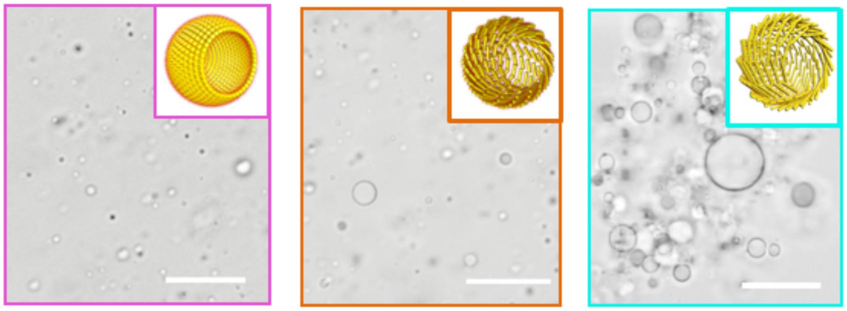

b

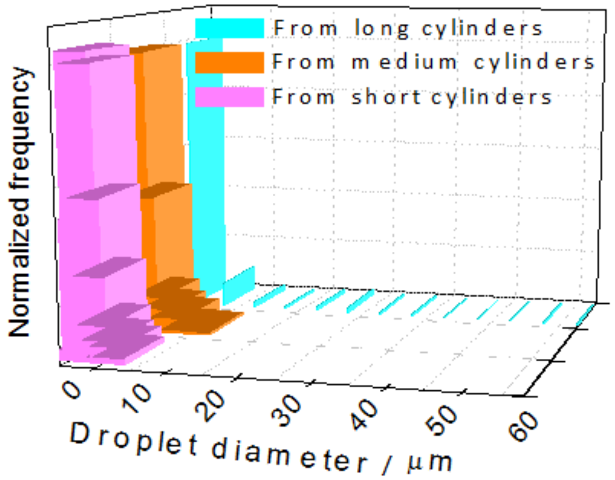

Supplementary Figure 5 | Optical microscopy images (a) and corresponding contour diameter distribution (b) of water-in-oil Pickering emulsions prepared from size-specific BCP1 cylindrical micelles with L_n values of 43, 320, and 1129 nm. All samples were prepared at BCP1 concentration of 0.5 mg mL^{-1} and water/oil volume fractions $\phi_w = 0.05$. Scale bars, 20 µm.

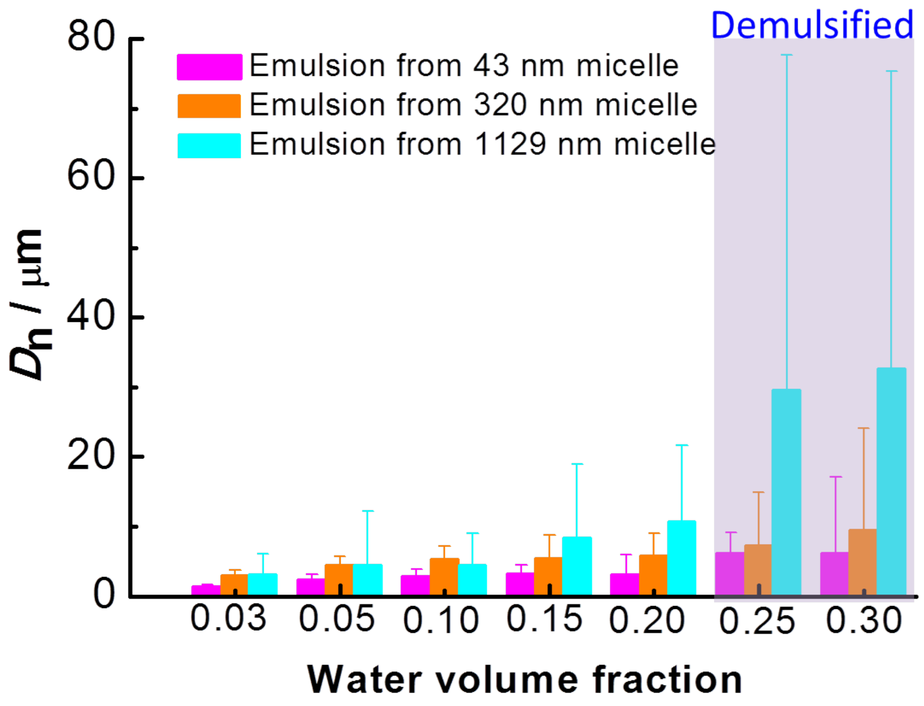

Supplementary Figure 6 | Plot of mean diameter (D_n) (bars) of water-in-oil Pickering emulsions droplets against water/oil volume fractions ϕ_w for samples prepared from BCP1 cylinder micelles with lengths, L_n = 43, 320, and 1129 nm. All samples were prepared at a BCP concentration of 0.5 mg mL^{-1}.

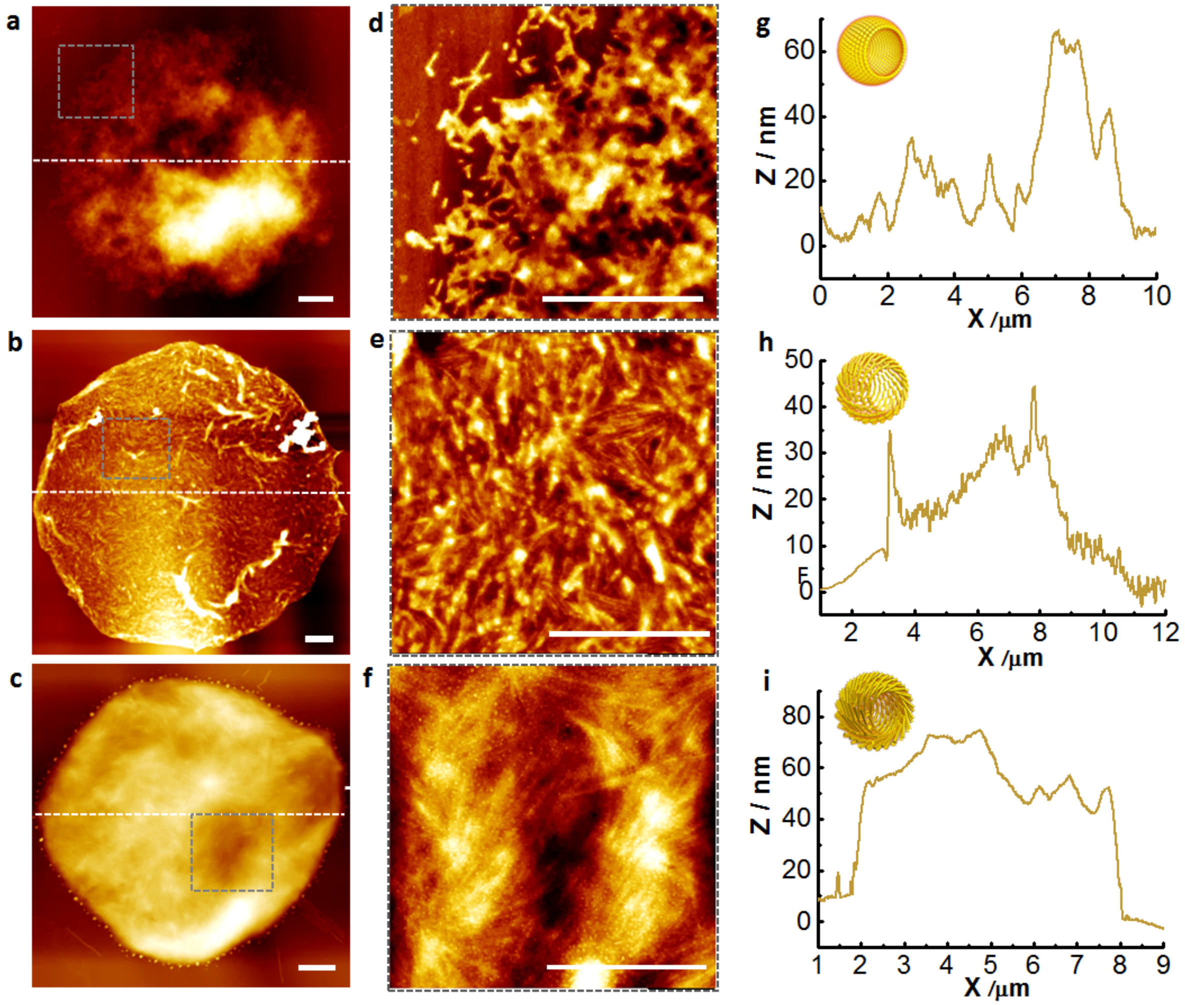

Supplementary Figure 7 | AFM images (a-f) and corresponding profiles (g-i) of air-dried partially collapsed and crumpled Pickering emulsion droplets prepared from BCP1 cylindrical micelles with lengths of 43 (a), 320 (b) and 1129 nm (c). (**d-f**) show high magnification images of areas delineated by the dashed grey squares in (**a-c**). (**g-i**) show height profiles along the dashed white lines highlighted in (**a-c**). Scale bars, 1 µm.

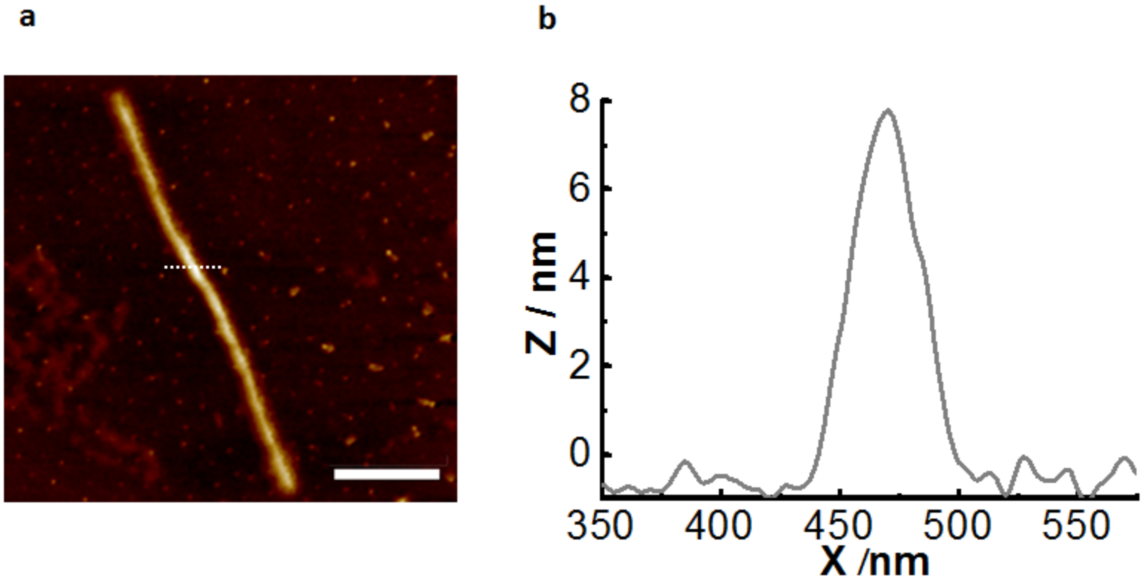

Supplementary Figure 8 | AFM image (a) and corresponding height profile (b) of a single BCP1 cylindrical micelle with a length of 850 nm. Corresponding height profile (**b**) along the dotted white line shown in (**a**) indicating that the micelle is *ca*. 8 nm in hight. The cylindrical micelles were prepared by the seeded growth of BCP1 in EHOH and then air-dried over 48 h onto cleaved mica. Scale bar, 200 nm.

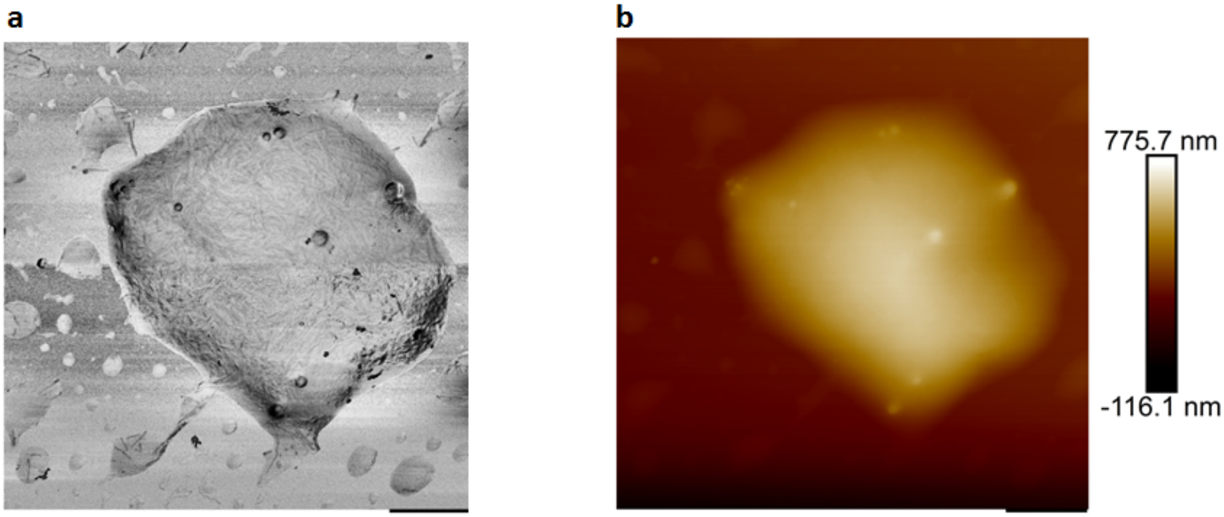

Supplementary Figure 9 | AFM elastic modulus mapping (a) and corresponding height image (b) of a single BCP1 Pickering emulsion droplet prepared from cylindrical micelles 320 nm in length. The sample was drop-cast and dried on a freshly cleaved mica sheet. Scale bars, 1 µm.

a

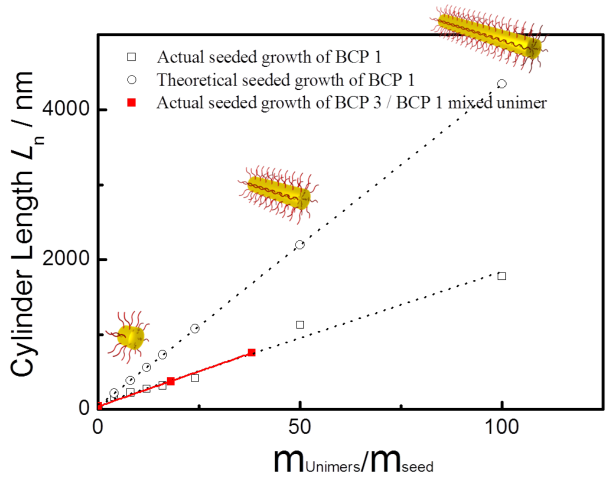

b

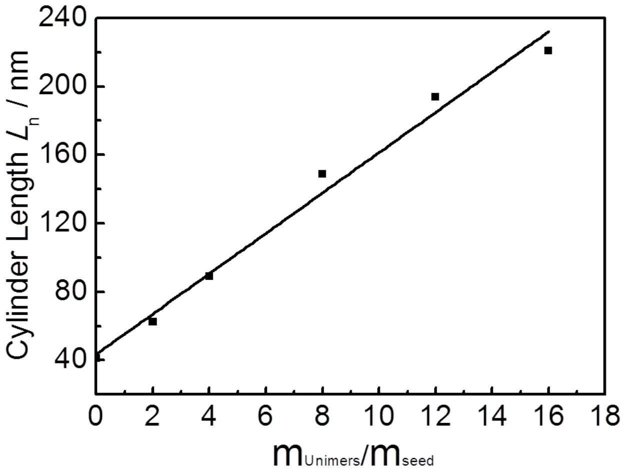

Supplementary Figure 10 | Plot of micelle length against $m_{unimers}/m_{seed}$ for seeded growth in EHOH (a) and isopropanol (b) in the presence of BCP1 or BCP 3/BCP 1 mixed unimers. (**a**) Plot showing theoretical and experimental linear dependence of micelle length (L_n) on the unimer : seed mass ratio ($m_{unimers}/m_{seed}$) for seeded growth in EHOH in the presence of BCP 1 or mixtures of BCP3/BCP1 unimers (m_{BCP3}/m_{BCP1} = 1 : 7). The seed micelles were 43 nm in length (L_w/L_n = 1.05). (**b**) Plot of micelle length against $M_{unimers}/M_{seed}$ for seeded growth in isopropanol in the presence of BCP1 unimers. Seed micelles were 41 nm in length (L_w/L_n = 1.06). The discrepancy in growth length between theory and experiment can be explained by incomplete unimer consumption at the point of workup or if the core associated with the new growth emanating from the seed has a slightly larger diameter where the chain folding is reduced. A similar discrepancy in the length of the newly grown region of cylindrical micelles from the predicted value has been observed in the seeded growth study of polythiophene containing block copolymers.[S4,S5]

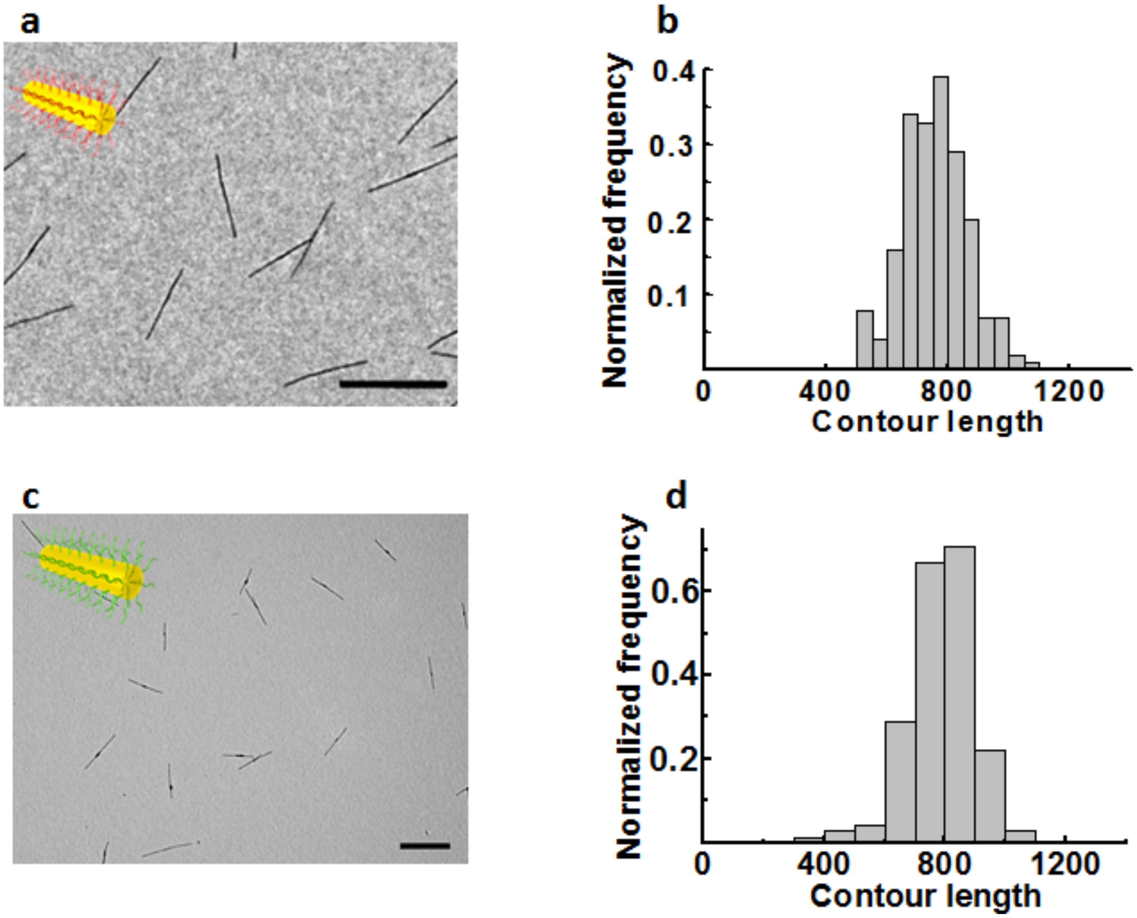

Supplementary Figure 11 | TEM micrographs (a,c) and corresponding contour length distribution (b,d) of red and green fluorescent cylindrical micelles. (**a**) TEM micrograph and (**b**) corresponding contour length distribution of BCP1/BCP2 red fluorescent cylindrical micelles (L_n = 756 nm, L_w/L_n = 1.02). (**c**) TEM micrograph and (**d**) corresponding contour length distribution of green fluorescent cylindrical micelles (L_n = 789 nm, L_w/L_n = 1.02) prepared from BCP1/BCP3. Scale bars, 1 μm.

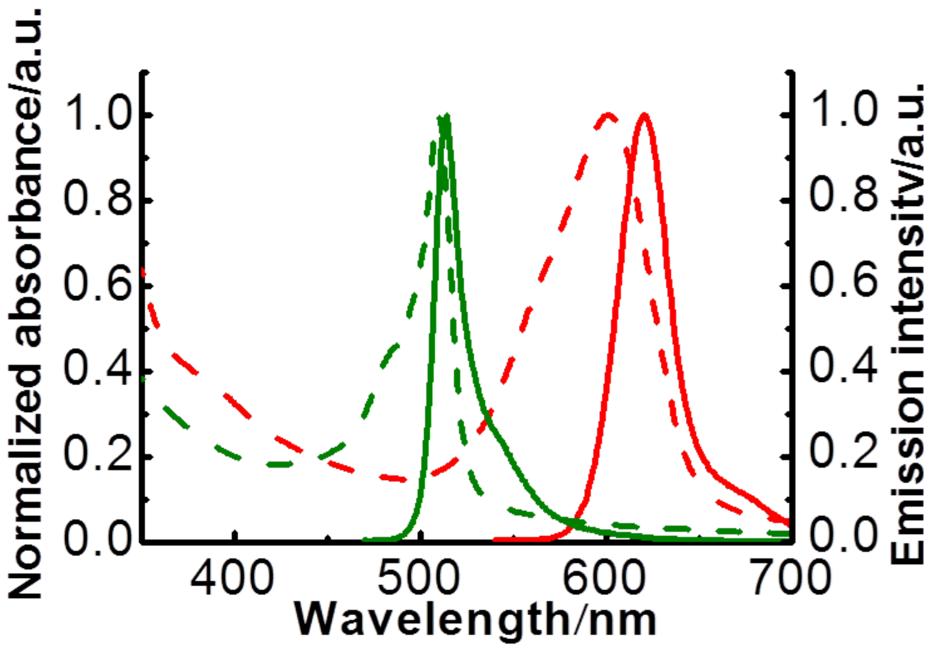

Supplementary Figure 12 | Normalized absorption (dashed line) and emission (solid line) fluorescence spectra of BCP1/BCP2 (red plots) and BCP1/BCP3 (green plots) cylindrical micelles dispersed in EHOH.

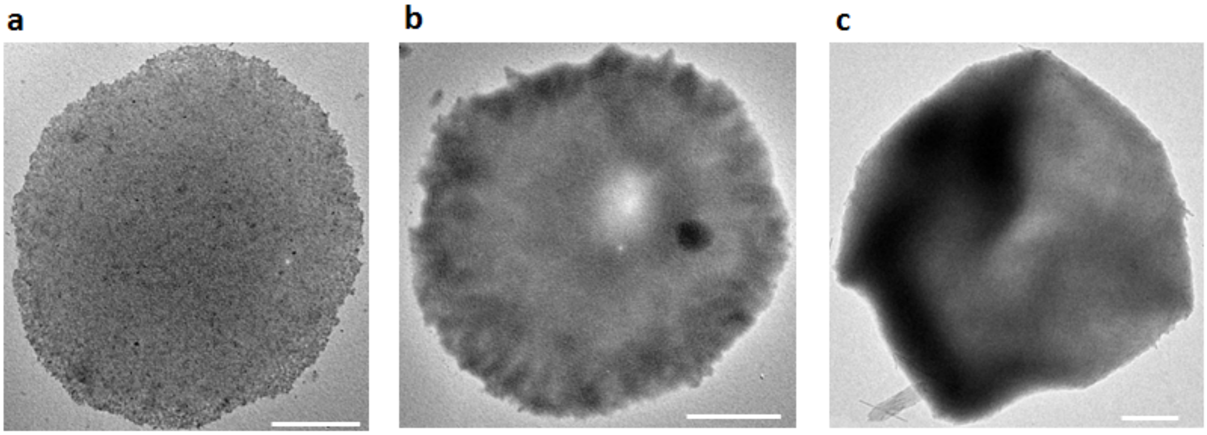

Supplementary Figure 13 | TEM images of air-dried cross-linked colloidosomes dispersed in isopropanol continuous phase. TEM images of air-dried cross-linked colloidosomes prepared from the 43 nm-sized micellar seeds (L_n = 43 nm, L_w/L_n = 1.05) (a), and 320 (L_n = 320 nm, L_w/L_n = 1.06) (b) or 1129 nm-long (L_n = 1129 nm, L_w/L_n = 1.02) (c) cylindrical micelles. The cross-linked colloidosomes were dispersed in an isopropanol continuous phase before being air-dried. Scale bars, 1 μm**.**

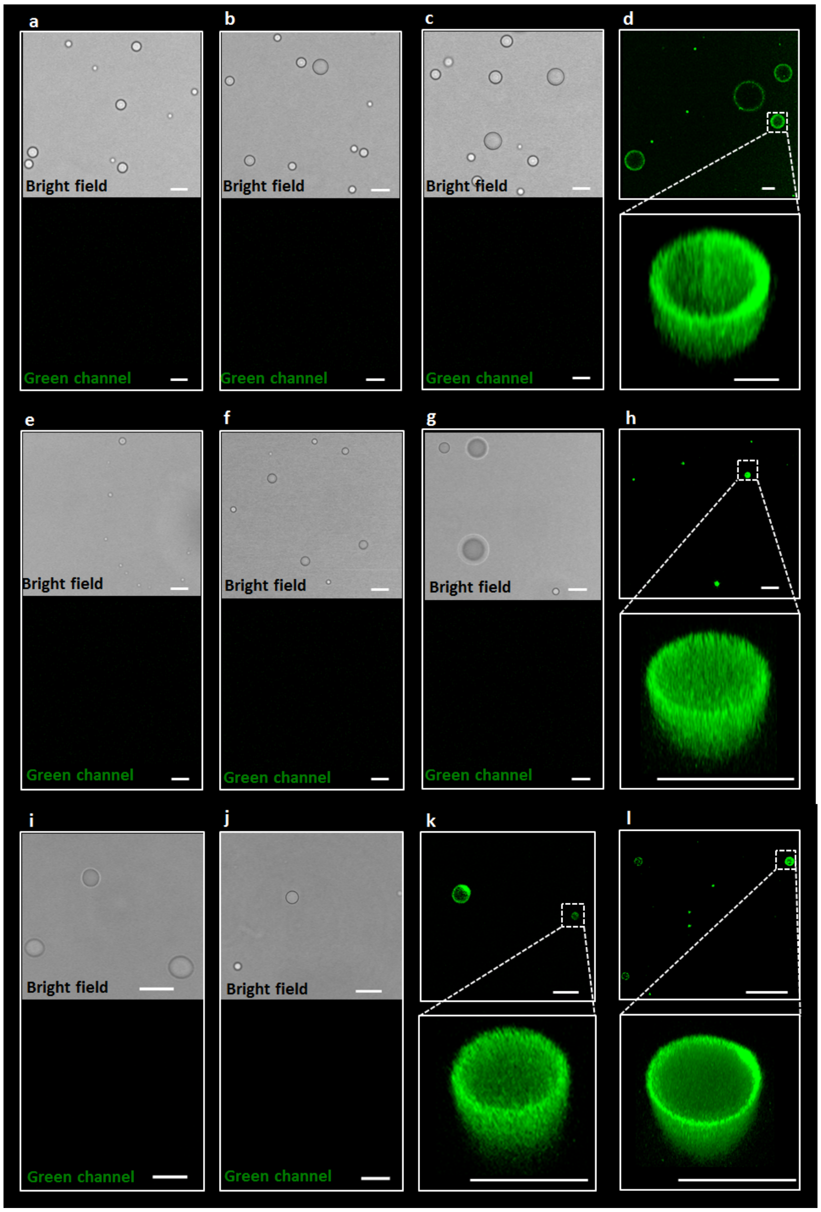

Supplementary Figure 14 | Confocal fluorescence micrographs of cross-linked BCP1 colloidosomes prepared with encapsulated FITC-dextran of different molecular weights; (a, e, and i) 4,400, (b, f, and j) 10,000, (c, g, and k) 70,000, and (d, h, and l) 150,000 Da. The colloidosomes were fabricated from 43 nm-sized micellar seeds (L_n = 43 nm, L_w/L_n = 1.05) (**a-d**), and 320 (L_n = 320 nm, L_w/L_n = 1.06) (**e-h**) or 882 nm-long (L_n = 882 nm, L_w/L_n = 1.01) (**i-l**) cylindrical micelles at a BCP 1 concentration of 0.5 mg mL^{-1} and ϕ_w = 0.05, and then dispersed in a water/isopropanol mixture (volume ratio, V_{water}/V_{iPrOH} = 4:1) to assess membrane permeability of the various polysaccharides. Absence of green fluorescence in (**a-c**), (**e-g**) and (**i-j**), and retained green fluorescence in (**d**), (**h**), (**k**), and (**l**) indicate that the low and high molecular weight polysaccharides are membrane permeable or impermeable, respectively. Scale bars in a-l, 10 μm, scale bars in the enlarged images of d, h, k and l are 5 μm.

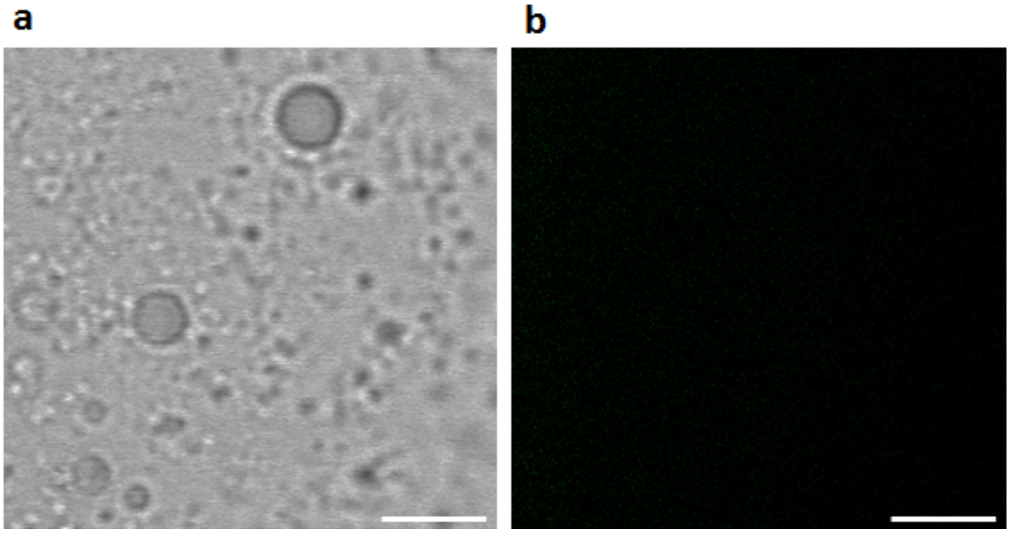

Supplementary Figure 15 | A control experiment involving a mixed solution of cross-linked BCP1 colloidosomes (fabricated from 882 nm-long cylindrical micelles) and FITC-dextran (molecular weight, 150,000 Da) showed an absence of green fluorescence in association with the colloidosomes. (**a**) and (**b**) are Confocal fluorescence micrographs in bright field and fluorescent channel of a mixed solution of cross-linked BCP1 colloidosomes and FITC-dextran (molecular weight, 150,000 Da). The colloidosomes were fabricated from 882 nm-long (L_n = 882 nm, L_w/L_n = 1.01) cylindrical micelles at a BCP 1 concentration of 0.5 mg mL^{-1} and ϕ_w = 0.05, and then dispersed in a water/isopropanol mixture (volume ratio, V_{water}/V_{iPrOH} = 4:1). Absence of green fluorescence in association with the colloidosomes in (**b**) indicates that there is no specific interaction between the cylindrical micelles and FITC-dextran molecules. Scale bars, 5 μm.

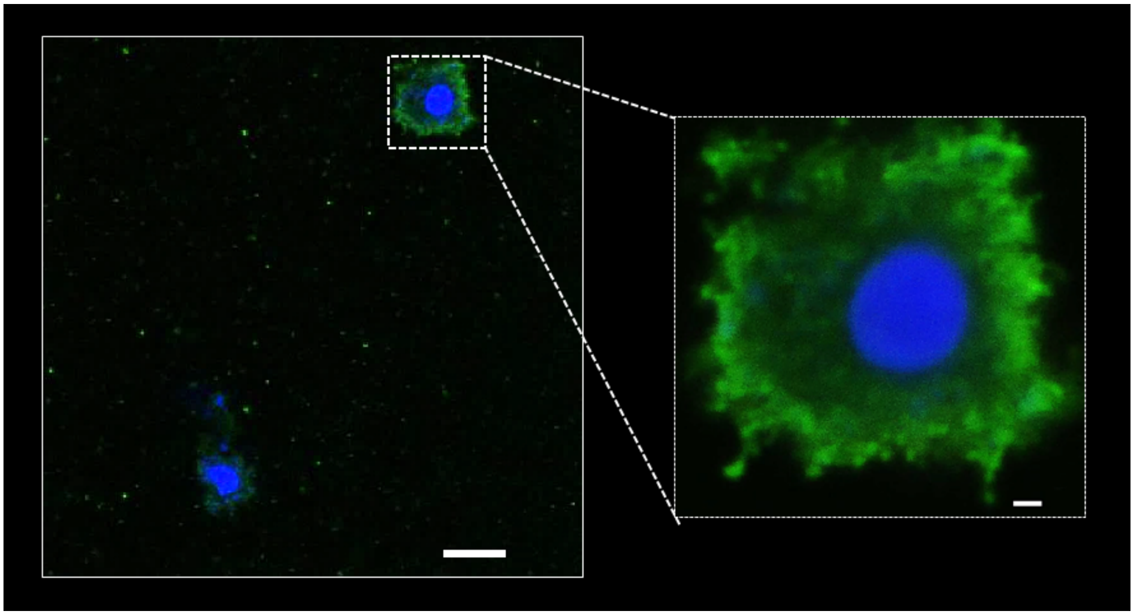

Supplementary Figure 16 | Confocal fluorescence microscopy micrographs recorded 20 min after mixing an isopropanol dispersion of blue dextran-containing cross-linked BCP1 colloidosomes (L_n = 882 nm, L_w/L_n = 1.01) with a THF solution of BCP1/BCP3 mixed unimers (mass ratio 7/1). The presence of a fluorescent outer shell in the partially collapsed colloidosomes indicates epitaxially elongation of the BCP1 cylindrical micelles at the surface of the membrane. Scale bar, 10 µm. Scale bar in the enlarged image is 1 µm.

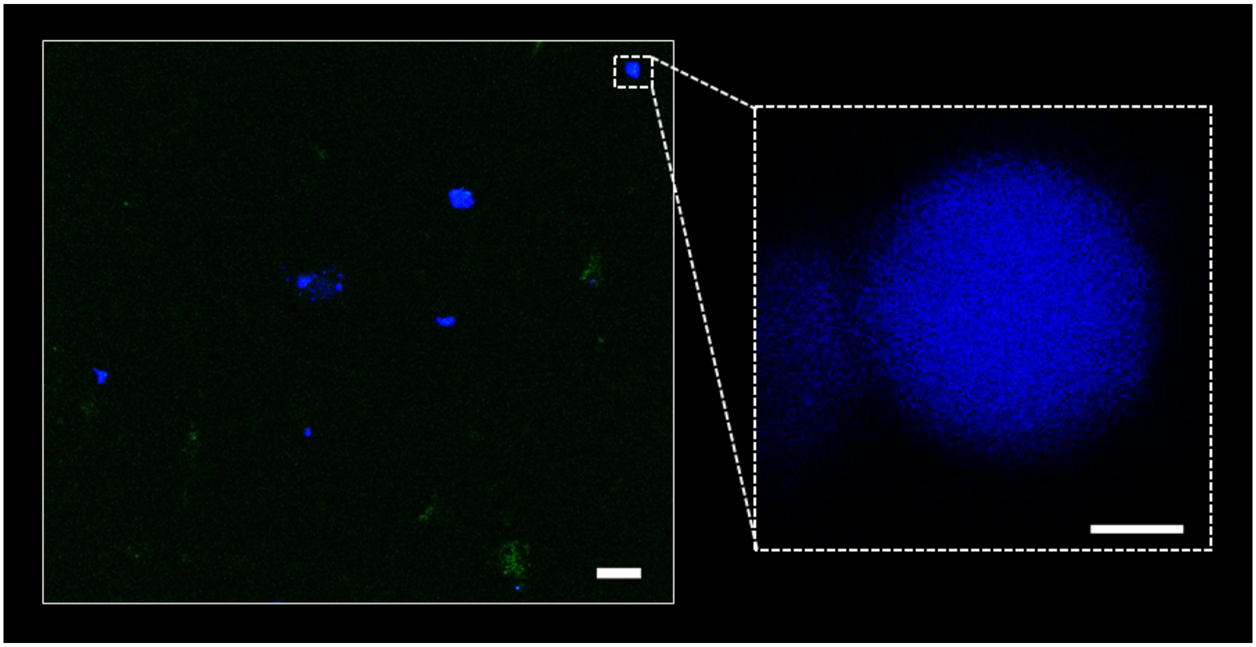

Supplementary Figure 17 | Confocal fluorescence microscopy micrographs of control samples prepared by mixing an isopropanol dispersion of blue dextran-containing cross-linked BCP1 colloidosomes (L_n = 882 nm, L_w/L_n = 1.01) with an isopropanol dispersion of BCP3 cylinders (L_n = 789 nm, L_w/L_n = 1.02). No green fluorescence is associated with the colloidosomes. Scale bar, 10 μm. Scale bar in the enlarged image is 1 μm.

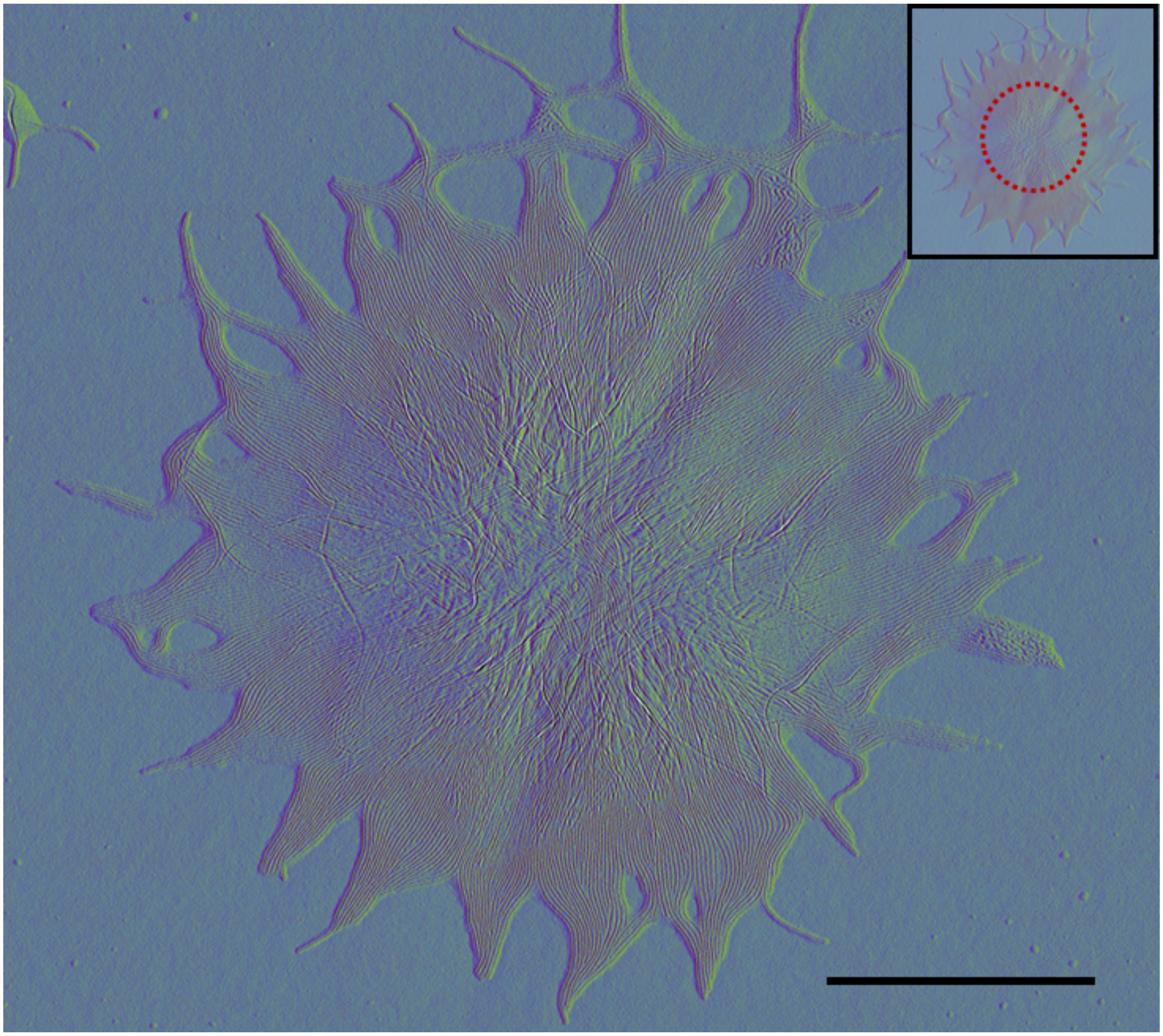

Supplementary Figure 18 | A map of Peak force AFM error signal of a partially collapsed cross-linked colloidosome with hair-like membrane texture produced by sequential addition of mixtures of BCP1/BCP2 and BCP1/BCP3 unimers to a dispersion of cross-linked BCP1 colloidosomes. The error signal emphasizes the fine structure of the membrane. Inset; corresponding low magnification image showing approximate diameter of the native colloidosome (red circle). Scale bar, 2 µm.

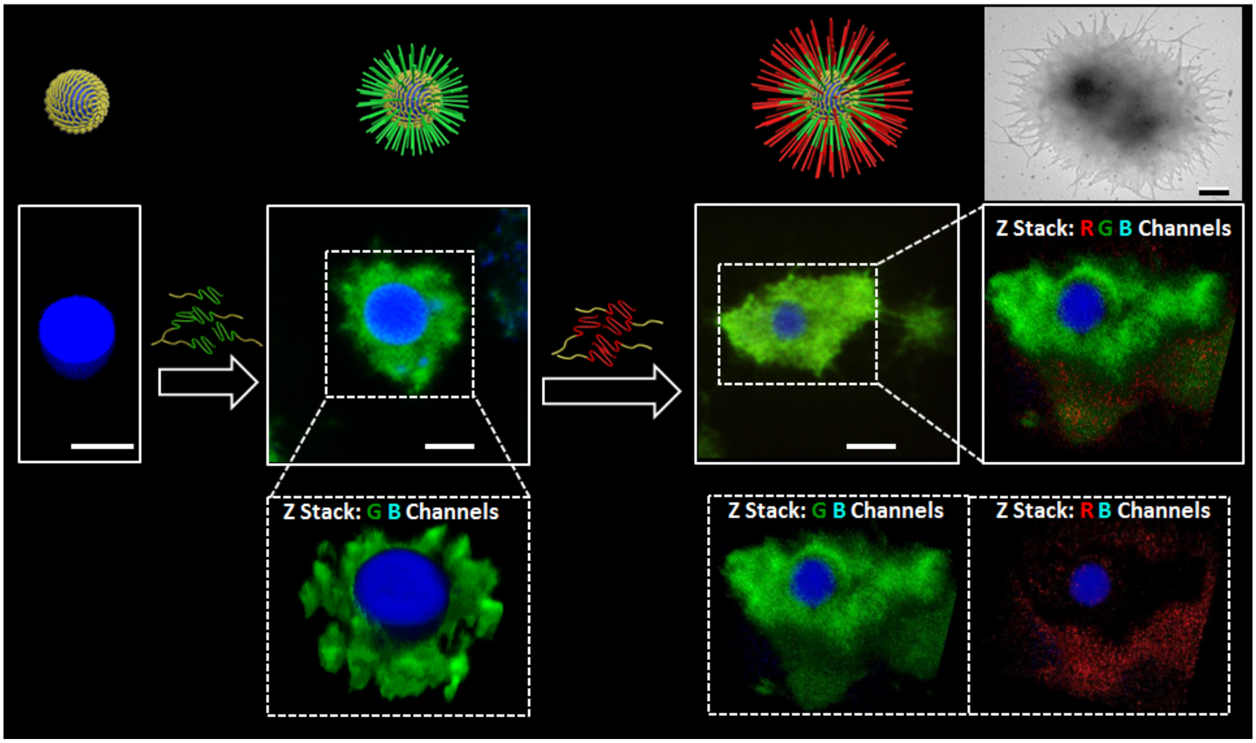

Supplementary Figure 19 | Structural and compositional elaboration of cross-linked BCP1 colloidosomes by a two-step sequence of epitaxial attachment of BCP1/BCP3 (green) and BCP1/BCP2 (red) unimers at the membrane surface. Schematic representations of the microstructures are shown along the top row. Confocal fluorescence microscopy images of cross-linked colloidosomes prepared by interfacial assembly of Cascade blue-dextran-containing size-specific (L_n = 882 nm, L_w/L_n = 1.01) BCP1 cylindrical micelles are shown in the middle row along with insets. Samples were prepared at a unimer : colloidosome mass ratio of 7 : 1. Under these conditions, the colloidosomes exhibited an extensively modified membrane. Scale bars in confocal microscope images 5 μm. Scale bar in TEM image, 1 μm.

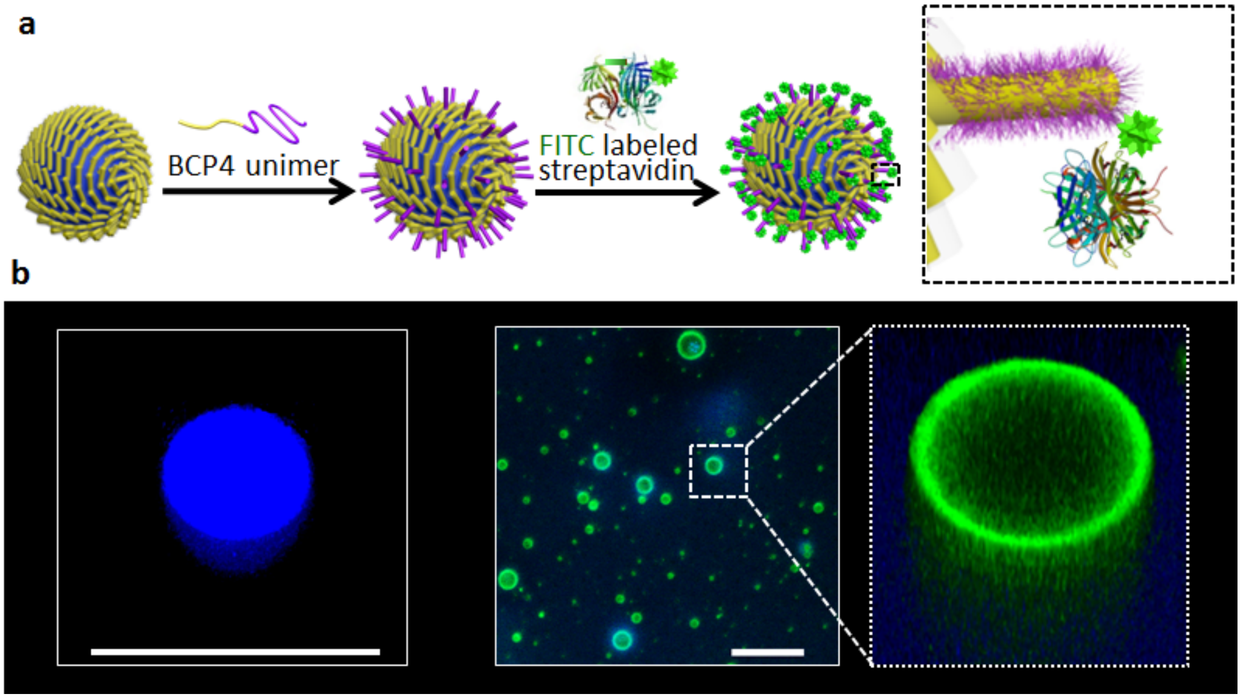

Supplementary Figure 20 | Schematic representation (a) and confocal fluorescence microscopy images (b) showing preparation of biotinylated colloidosomes and membrane binding of FITC-streptavidin. Confocal fluorescence microscopy images in (**b**) show cross-linked blue dextran-containing colloidosomes prepared by interfacial assembly of size-specific (L_n = 882 nm, L_w/L_n = 1.01) BCP1 cylindrical micelles; the left image shows a blue fluorescent interior in the native colloidosome confirming initial presence of encapsulated polysaccharide (M_w = 10,000); middle and right images illuminated at blue and green channels show colloidosomes after addition of BCP1/BCP4 unimers and subsequent conjugation of FITC-streptavidin. Leakage of the encapsulated dextran is observed due to the relatively low molecular weight of the polysaccharide. Scale bars, 10 μm.

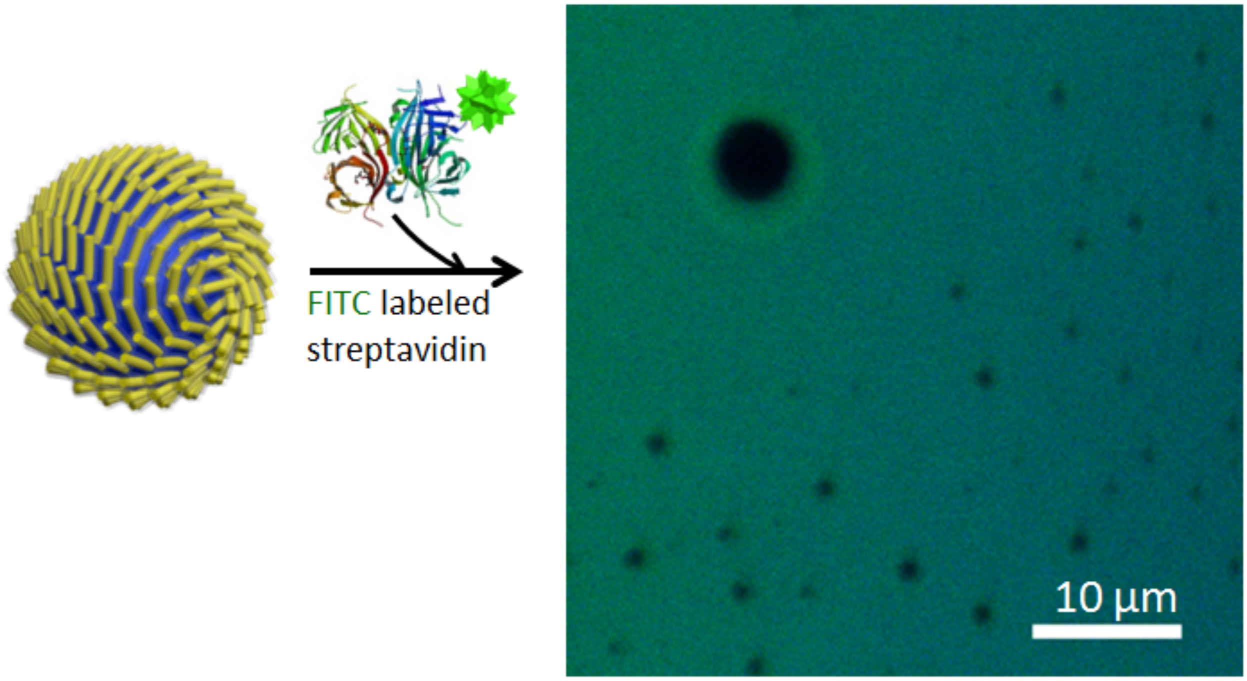

Supplementary Figure 21 | Schematic illustration and confocal fluorescence micrograph of a control dispersion of unmodified (non-biotinylated) BCP1-based colloidosomes (L_n = 882 nm, L_w/L_n = 1.01) in the presence of streptavidin. No interactions between the components are observed. Samples were prepared at a water/isopropanol volume ratio of 4 : 1. Scale bar, 10 µm.

Supplementary References

1. Gilroy, J. B. *et al.* Monodisperse cylindrical micelles by crystallization-driven living self-assembly. *Nature Chem.* **2**, 566-570 (2010).
2. Finnegan, J. R. *et al.* Gradient Crystallization-Driven Self-Assembly: Cylindrical Micelles with "Patchy" Segmented Coronas via the Coassembly of Linear and Brush Block Copolymers. *J. Am. Chem. Soc.* **136**, 13835-13844 (2014).
3. Gadt, T., Ieong, N. S., Cambridge, G., Winnik, M. A. & Manners, I. Complex and hierarchical micelle architectures from diblock copolymers using living, crystallization-driven polymerizations. *Nature Mater.* **8**, 144-150 (2009).
4. Gwyther, J. *et al.* Dimensional Control of Block Copolymer Nanofibers with a -Conjugated Core: Crystallization-Driven Solution Self-Assembly of Amphiphilic Poly(3-hexylthiophene)-b-poly(2-vinylpyridine). *Chem.-Eur. J.* **19**, 9186-9197 (2013).
5. S. K. Patra, R. Ahmed, G. R. Whittell, D. J. Lunn, E. L. Dunphy, M. A. Winnik, I. Manners, Cylindrical Micelles of Controlled Length with a π-Conjugated Polythiophene Core via Crystallization-Driven Self-Assembly. *J. Am. Chem. Soc.,* **133**, 8842-8845 (2011).
